# Emerging Gel Technologies for Atherosclerosis Research and Intervention

**DOI:** 10.3390/gels12010080

**Published:** 2026-01-16

**Authors:** Sen Tong, Jiaxin Chen, Yan Li, Wei Zhao

**Affiliations:** 1 Yunnan Key Laboratory of Integrated Traditional Chinese and Western Medicine for Chronic Disease in Prevention and Treatment, Yunnan University of Chinese Medicine, Kunming 650500, China; tongsen@ynucm.edu.cn (S.T.); chenjiaxin@ynucm.edu.cn (J.C.); 2 Faculty of Life Science and Technology, Kunming University of Science and Technology, Kunming 650500, China; liyanken@126.com

**Keywords:** hydrogels, atherosclerosis, drug delivery systems, nanomedicine, theranostic nanomedicine, in vitro techniques, biocompatible materials

## Abstract

Atherosclerosis remains a leading cause of cardiovascular mortality despite advances in pharmacological and interventional therapies. Current treatment approaches face limitations including systemic side effects, inadequate local drug delivery, and restenosis following vascular interventions. Gel-based technologies offer unique advantages through tunable mechanical properties, controlled degradation kinetics, high drug-loading capacity, and potential for stimuli-responsive therapeutic release. This review examines gel platforms across multiple scales and applications in atherosclerosis research and intervention. First, gel-based in vitro models are discussed. These include hydrogel matrices simulating plaque microenvironments, three-dimensional cellular culture platforms, and microfluidic organ-on-chip devices. These devices incorporate physiological flow to investigate disease mechanisms under controlled conditions. Second, therapeutic strategies are addressed through macroscopic gels for localized treatment. These encompass natural polymer-based, synthetic polymer-based, and composite formulations. Applications include stent coatings, adventitial injections, and catheter-delivered depots. Natural polymers often possess intrinsic biological activities including anti-inflammatory and immunomodulatory properties that may contribute to therapeutic effects. Third, nano- and microgels for systemic delivery are examined. These include polymer-based nanogels with stimuli-responsive drug release responding to oxidative stress, pH changes, and enzymatic activity characteristic of atherosclerotic lesions. Inorganic–organic composite nanogels incorporating paramagnetic contrast agents enable theranostic applications by combining therapy with imaging-guided treatment monitoring. Current challenges include manufacturing consistency, mechanical stability under physiological flow, long-term safety assessment, and regulatory pathway definition. Future opportunities are discussed in multi-functional integration, artificial intelligence-guided design, personalized formulations, and biomimetic approaches. Gel technologies demonstrate substantial potential to advance atherosclerosis management through improved spatial and temporal control over therapeutic interventions.

## 1. Introduction

Atherosclerosis remains a leading cause of cardiovascular morbidity and mortality worldwide, characterized by progressive accumulation of lipids, inflammatory cells, and extracellular matrix within arterial walls [1,2]. Traditional pharmacological approaches include statins, antiplatelet agents, and antihypertensive medications. These therapies address systemic risk factors but demonstrate limited efficacy in reversing established plaques [3]. Surgical interventions such as bypass grafting provide mechanical revascularization yet carry substantial procedural risks and complications. Percutaneous coronary interventions include balloon angioplasty and stent implantation. These approaches have revolutionized acute treatment by restoring blood flow through stenotic vessels [4]. However, bare metal stents frequently induce restenosis through excessive neointimal hyperplasia, while drug-eluting stents releasing antiproliferative agents have reduced but not eliminated this complication [5]. Current stent coatings face several limitations. Burst release kinetics, polymer-induced inflammation, and delayed endothelial healing predispose to late thrombosis [6]. These challenges motivate development of advanced biomaterial platforms. Such platforms enable more precise spatial and temporal control over therapeutic agent delivery to diseased vessel segments. They also promote favorable vascular healing responses.

Gel-based technologies offer unique advantages for both investigating atherosclerosis mechanisms and developing localized treatment strategies. Hydrogels are three-dimensional crosslinked polymer networks containing substantial water content. They provide tunable mechanical properties, controlled degradation kinetics, and high drug-loading capacity [7]. These platforms span multiple length scales with distinct pharmacological applications. Macroscopic hydrogels function as millimeter-scale depot formulations applied directly at diseased sites [8]. Three primary delivery modes exist for these applications. Stent coatings provide thin conformal layers on metallic scaffolds releasing drugs during and after device implantation. Balloon coatings enable brief contact delivery during angioplasty inflation transferring therapeutics into arterial walls. Adventitial injections place gel depots around vessel exteriors avoiding luminal exposure. These localized delivery approaches enable sustained drug release at specific vascular sites while minimizing systemic exposure. Nanogels are submicron crosslinked polymer networks typically ranging from fifty to two hundred nanometers [9]. They circulate systemically following intravenous administration. Their colloidal dimensions permit extravasation through dysfunctional endothelium. They can accumulate at atherosclerotic plaques through passive targeting or active recognition via surface ligand modifications. Microgels represent an intermediate scale between macroscopic depots and nanogels. These particles range from one to one hundred micrometers. They are employed in specialized applications including flow-directed embolization of plaque neovascularization or ultrasound-triggered drug release. Beyond therapeutic applications, gel matrices provide physiologically relevant three-dimensional environments for cell culture and tissue engineering. These platforms enable investigation of plaque microenvironment effects on cellular behaviors under controlled conditions. They bridge the gap between conventional two-dimensional culture and animal models [10].

Recent reviews have addressed various aspects of biomaterial applications in cardiovascular disease. A 2025 review by Gu et al. examined nanoparticle-based therapies for atherosclerosis, focusing on systemic delivery without covering macroscopic depots or modeling platforms [11]. Chen and colleagues surveyed in vitro atherosclerosis models, discussing culture systems and microfluidic devices but not therapeutic applications [12]. Work from Guan’s group reviewed stimuli-responsive hydrogels for cardiovascular repair across multiple diseases including myocardial infarction [13]. Grimaudo et al. examined nanogels in regenerative medicine spanning various tissues [14]. These reviews typically focus on either investigation tools or therapeutic delivery within specific scale ranges. Atherosclerosis management would benefit from integrated consideration across multiple scales. The present review addresses this gap by examining gel technologies spanning in vitro modeling platforms, macroscopic therapeutic depots, and nanoscale delivery vehicles. This multi-scale perspective provides comparative discussion guiding material selection for specific atherosclerosis applications.

The versatility of gel platforms arises from diverse polymer chemistries and multiple strategies for controlling network formation and degradation [15]. Natural polymers include polysaccharides such as hyaluronic acid, alginate, and chitosan. These materials provide inherent biocompatibility and biological recognition through specific receptor interactions [16]. Protein-derived materials including collagen and peptide-based hydrogels offer cell adhesion motifs and protease-sensitive degradation matching native extracellular matrix properties. Synthetic polymers include polyethylene glycol and polyvinyl alcohol. These materials enable precise molecular weight control. They also permit incorporation of stimuli-responsive groups responding to oxidative stress, pH changes, or enzymatic activity characteristic of atherosclerotic lesions [17]. Composite approaches combine natural and synthetic polymers or incorporate functional nanoparticles. These strategies extend capabilities to include catalytic antioxidant activity, magnetic responsiveness, or imaging contrast for theranostic applications.

This review covers gel-based technologies for atherosclerosis research and intervention across multiple scales and application contexts. First, gel-based in vitro models are discussed. These include hydrogel matrices simulating plaque microenvironments, three-dimensional cellular culture platforms, and microfluidic organ-on-chip devices. Such devices incorporate controlled flow to investigate disease mechanisms under physiologically relevant conditions. Second, gel-based therapeutic strategies are addressed. Macroscopic gels for localized treatment encompass natural polymer-based, synthetic polymer-based, and composite formulations. Nano- and microgels for systemic delivery include polymer-based and inorganic–organic composite nanogels with imaging capabilities. Third, a comparative discussion evaluates material selection tradeoffs across different gel platforms and application requirements. Finally, current challenges are discussed. These include manufacturing consistency, long-term safety validation, and regulatory pathway definition. Future opportunities are also addressed. These encompass multi-functional integration, artificial intelligence-guided design, personalized formulations, and biomimetic approaches. Throughout this review, emphasis is placed on rational design of material properties to address specific pathophysiological features. Inherent limitations in model systems and emerging therapeutic platforms are also recognized.

## 2. Gel-Based In Vitro Models for Atherosclerosis Research

### 2.1. Hydrogel Matrices for Plaque Microenvironment Simulation

**Figure 1:**
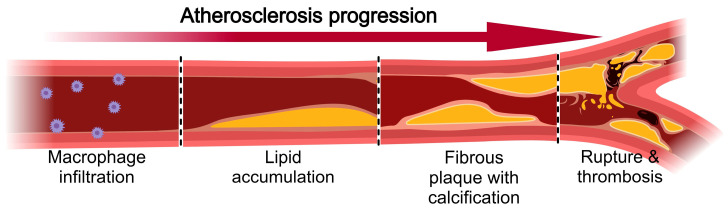
Atherosclerosis progression from initial lesion to complicated plaque rupture. This figure was created with MedPeer (https://image.medpeer.cn/, accessed on 10 January 2026).

Atherosclerosis progresses through distinct pathological stages that hydrogel models aim to recapitulate (Figure 1). Disease initiation involves endothelial dysfunction triggered by risk factors including hyperlipidemia, hypertension, and inflammatory mediators [18]. Dysfunctional endothelium exhibits increased permeability, allowing low-density lipoprotein penetration into subendothelial space. Retained lipoproteins undergo oxidative modification, attracting circulating monocytes that differentiate into macrophages. These macrophages internalize modified lipoproteins forming lipid-laden foam cells visible as fatty streaks. Continued lipid accumulation generates extracellular lipid pools within vessel walls [19]. Smooth muscle cells migrate from media into intima and proliferate while secreting collagen and proteoglycans that form fibrous caps overlying lipid cores. Advanced lesions develop necrotic cores containing cholesterol crystals, cellular debris, and extracellular matrix fragments. Calcification occurs through deposition of hydroxyapatite crystals in regions of necrosis and matrix remodeling. Vulnerable plaques characterized by large lipid cores, thin fibrous caps, and inflammatory cell infiltration become susceptible to rupture [20]. Cap disruption exposes thrombogenic materials triggering platelet aggregation and coagulation cascade activation. Resulting thrombi can partially or completely occlude arterial lumens causing acute ischemic events.

Atherosclerotic plaques develop through complex cellular and matrix interactions that traditional two-dimensional culture systems cannot adequately replicate. Hydrogel matrices address this limitation by providing three-dimensional scaffolds whose composition, mechanical properties, and degradability can be tuned to mimic specific features of diseased arterial tissue [21]. The native arterial wall consists primarily of collagen providing tensile strength, elastin enabling elastic recoil, and proteoglycans contributing to water retention and compressive resistance. During atherosclerosis, this matrix undergoes substantial remodeling including collagen accumulation, elastin fragmentation, and altered stiffness profiles. Hydrogel platforms enable investigation of how these microenvironmental changes influence cellular behaviors by allowing independent control of mechanical and biochemical variables [22]. Collagen hydrogels represent physiologically relevant matrices due to collagen’s predominance in fibrous caps that stabilize plaques. Type I collagen self-assembles into fibrillar networks through pH and temperature-mediated mechanisms, generating structures whose stiffness depends on polymer concentration and gelation conditions [23]. These matrices prove particularly valuable for studying vascular calcification, where extracellular vesicles secreted by vascular cells serve as mineral nucleation sites [24]. For example, recent work employing three-dimensional collagen hydrogels incubated with calcifying vesicles demonstrated that bisphosphonates alter microcalcification size and morphology in time-dependent patterns. When these drugs are added before vesicle aggregation initiates, the resulting mineral deposits exhibit reduced size and modified crystal structure. Computational modeling indicates that drug treatment alters calcification patterns in ways that reduce mechanical stress concentrations within fibrous caps [25]. Another study revealed strong interdependence between collagen density and mineral deposition patterns [26]. This investigation revealed strong interdependence between collagen density and mineral deposition patterns. Higher collagen concentrations constrain vesicle mobility. This promotes formation of dispersed small calcifications rather than fewer large deposits. These observations suggest implications for plaque stability, as computational modeling indicates that numerous small calcifications generate more uniform stress distributions within fibrous caps compared to fewer large calcifications that create stress concentrations.

Synthetic polymer hydrogels based on polyvinyl alcohol provide mechanical stability and imaging capabilities complementary to collagen matrices [27]. Polyvinyl alcohol forms physically crosslinked networks with high mechanical strength and enzymatic resistance. These properties enable long-term stability for applications requiring repeated testing or extended observation periods [28]. Recent studies have demonstrated the utility of polyvinyl alcohol for creating patient-specific atherosclerotic plaque phantoms that enable validation of emerging imaging modalities [29]. These materials can mimic the mechanical properties of different plaque components ranging from soft lipid pools to stiff calcifications. These phantoms enable validation of imaging algorithms by comparing measured properties against known mechanical values. Patient-specific geometries can be created from magnetic resonance imaging data. These phantoms maintain consistent mechanical properties over extended periods, enabling quality control across different imaging systems. Polyvinyl alcohol hydrogels also enable creation of intracranial atherosclerotic models with accurate geometry and tissue-mimicking properties [30]. Phantom scans across multiple imaging sites show high reproducibility, confirming dimensional stability under repeated scanning.

**Figure 2:**
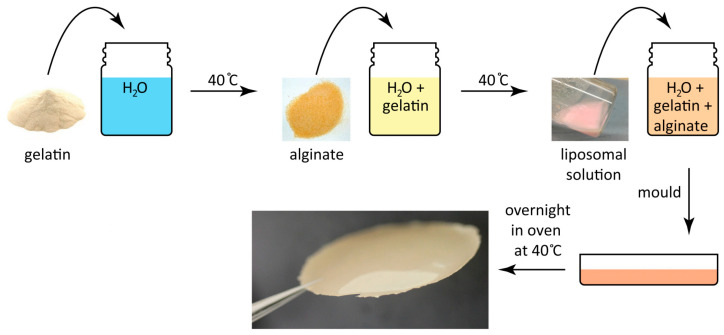
Schematic illustration of artificial atherosclerotic plaque fabrication through sequential mixing of gelatin, alginate, and liposomal lipid solutions followed by molding and calcium chloride crosslinking. Reproduced with permission from reference [31]. Open access.

Composite hydrogel systems combining gelatin and alginate enable fabrication of artificial plaques with spatially heterogeneous composition. Razzi and colleagues developed implantable lipid-rich hydrogels that mimic type IV atherosclerotic lesions to investigate drug transport from stents into diseased arterial walls [31]. The fabrication involves dissolving gelatin in heated water, incorporating sodium alginate, and mixing in liposomes containing physiologically relevant cholesterol and phospholipid concentrations before molding and crosslinking with calcium chloride (Figure 2). These artificial plaques were sutured onto everolimus-eluting stents and implanted in porcine coronary arteries for ex vivo perfusion experiments. Mass spectrometry imaging demonstrated that lipid-rich plaques significantly reduced drug penetration into arterial tissue compared to lipid-free controls. This finding confirms that atherosclerotic plaque composition substantially affects therapeutic drug distribution. The model provides a reproducible platform for systematic studies of plaque–drug interactions in coronary disease.

These hydrogel platforms provide experimentally tractable conditions for investigating atherosclerosis mechanisms. Natural matrices provide cell-instructive properties and enzymatic degradability, while synthetic polymers offer mechanical robustness for imaging applications. Composite approaches enable creation of structures mimicking multiple plaque components. The reductionist nature allows systematic variation in individual parameters to explore potential causal relationships, though simplified models cannot fully recapitulate the cellular heterogeneity and systemic influences present in vivo.

### 2.2. Three-Dimensional Cellular Models in Gel Platforms

Three-dimensional gel platforms restore aspects of native tissue architecture that monolayer cultures lack. Cells on rigid plastic substrates experience artificial mechanical cues and exhibit gene expression patterns diverging from in vivo counterparts [32]. Embedding cells within gel matrices provides physiological extracellular matrix contacts. These platforms enable three-dimensional cell–cell interactions. They also allow mechanical force transmission through viscoelastic networks [33]. These platforms are valuable for studying foam cell formation, endothelial barrier function, and inflammatory cell recruitment where spatial organization critically influences outcomes.

**Figure 3:**
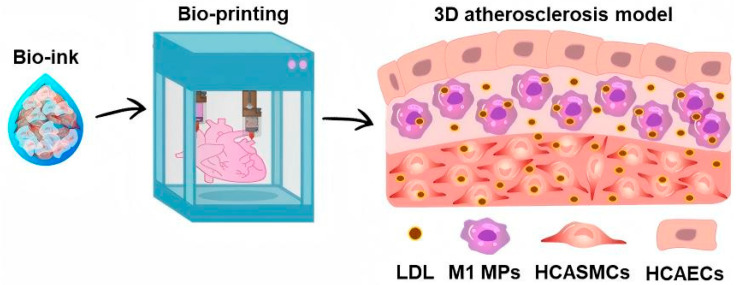
Schematic workflow of bioprinted atherosclerosis model fabrication using bioink containing multiple cell types and low-density lipoprotein treatment to induce disease progression. Reproduced with permission from reference [35]. Copyright 2025, IOP Publishing Ltd.

Endothelial barrier models require recapitulation of basement membrane structures separating endothelial cells from underlying pericytes. For example, porous polyvinyl alcohol hydrogels enable the co-culture of endothelial cells and pericytes in spatially defined configurations [34]. The gel separates the populations while permitting soluble factor diffusion. Endothelial cells develop adherens junctions and impermeable barriers that mature over several days. Nitric oxide secretion increases progressively to physiological levels. Hemoglobin alpha expression emerges through endothelial–pericyte signaling mediated by factors crossing the artificial membrane. When exposed to inflammatory stimuli and oxidized lipoproteins, the engineered endothelium exhibits increased permeability but subsequently recovers. Introducing monocytes into this system recapitulates foam cell formation. Monocytes adhere to activated endothelium and undergo transendothelial migration. They differentiate into macrophages that internalize oxidized lipoproteins. Another approach employs three-dimensional bioprinting with collagen methacryloyl to fabricate structures incorporating multiple cell types in organized arrangements. Kim and colleagues developed a three-dimensional bioprinted atherosclerosis model using collagen methacryloyl hydrogel containing inflammatory macrophages, smooth muscle cells, and endothelial cells [35]. Low-density lipoprotein treatment induced progressive atherosclerotic changes over fourteen days (Figure 3). Exosomes isolated from baseline, early atherosclerosis, and late atherosclerosis stages were characterized and analyzed using proteomics and microRNA sequencing. The bioprinted model successfully recapitulated key atherosclerotic features and provides a reproducible platform for biomarker discovery.

Macrophage foam cell formation within three-dimensional collagen gels enables the investigation of lipid metabolism and inflammatory processes. Recent studies show that collagen seeded with monocyte-derived macrophages and exposed to oxidized lipoproteins develops into necrotic core models [36]. The three-dimensional environment influences foam cell kinetics and cytokine profiles compared to monolayer cultures. Incorporating atorvastatin into collagen matrices inhibits foam cell formation and reduces pro-thrombotic properties. Quantitative coagulation assays demonstrate that foam cell-containing gels trigger rapid platelet aggregation, whereas drug treatment significantly reduces this response. Tissue factor pathway activity measurements confirm suppression of extrinsic coagulation initiation. This biomimetic neointimal layer provides a substrate for studying atherothrombosis mechanisms and screening antithrombotic candidates. Another study showed that chitosan hydrogels incorporating exosomes from nicotine-treated macrophages demonstrate paracrine signaling effects [37]. These exosomes are enriched in microRNA-21-3p and promote smooth muscle proliferation when released from gel depots. Implantation near vessels in rat aortic injury models increases neointimal thickness compared to control exosomes. Profiling identifies microRNA-21-3p as the mediator functioning through phosphatase and tensin homolog targeting.

These models show how gel scaffolds support the investigation of specific cell types under three-dimensional conditions. Co-culture configurations permit the study of cell–cell communication across tissue interfaces. The platforms are valuable for mechanistic pathway investigation and therapeutic screening. However, models remain simplified compared to plaque cellular complexity including multiple macrophage subsets, T lymphocytes, and dendritic cells. Static culture conditions also lack hemodynamic forces that critically influence endothelial function, limitations addressed by microfluidic platforms incorporating flow.

### 2.3. Microfluidic and Organ-on-Chip Gel Systems

Microfluidic platforms address limitations of static cultures by introducing controlled flow that mimics hemodynamic forces. Wall shear stress fundamentally regulates endothelial phenotype including morphology, gene expression, and inflammatory activation [38]. Laminar flow with physiological shear stress promotes endothelial quiescence. This phenotype is characterized by elongated morphology, elevated nitric oxide production, and suppressed adhesion molecule expression. Disturbed flow with low or oscillatory shear stress induces pro-inflammatory phenotypes [39]. Atherosclerotic plaques develop preferentially at arterial branch points and curvatures where disturbed flow creates low-shear-stress regions [40]. Static cultures cannot reproduce these flow-dependent phenomena. Microfluidic devices fabricated from transparent materials enable real-time observation of cellular responses, while gel matrices provide structural support and extracellular matrix interactions. Channel dimensions typically range from tens to hundreds of micrometers to generate physiological shear stress at achievable flow rates.

**Figure 4:**
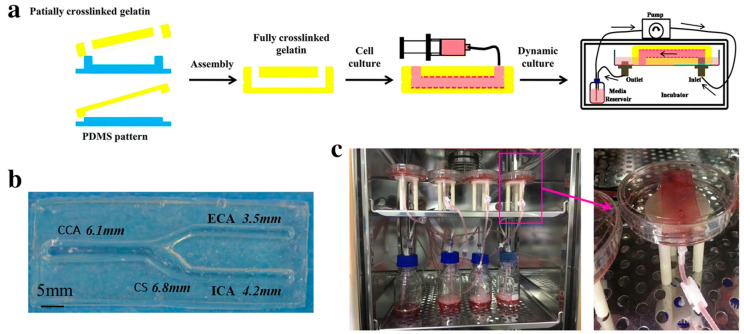
Fabrication workflow of the gelatin-based perfusable carotid artery model showing assembly of patterned hydrogel layers (**a**), endothelial cell seeding (**b**), and dynamic perfusion system setup (**c**). Reproduced with permission from reference [41]. Open access.

Recent studies demonstrate how gel-based microfluidic models recapitulate flow-dependent endothelial responses. Chen and colleagues engineered a gelatin-based perfusable carotid artery model to investigate wall shear stress effects on endothelial dysfunction in atherosclerosis [41]. The researchers fabricated a tuning fork-shaped structure using enzyme-crosslinked gelatin hydrogel with physiologically accurate dimensions. Computational fluid dynamics identified two regions with distinct flow patterns. The external carotid artery exhibited laminar flow with high wall shear stress, while the carotid sinus showed turbulent flow with low wall shear stress. Endothelial cells cultured under perfusion for twenty-four hours demonstrated progressive elongation in high shear regions but remained disorganized in low shear areas. Low shear stress significantly increased adhesion molecule expression and endothelial permeability. This model successfully recapitulated atherosclerosis-prone microenvironments (Figure 4). Another approach investigates how matrix composition influences flow responses using microchannels coated with collagen or fibronectin to represent healthy versus atherosclerotic phenotypes [42]. Cells on collagen align uniformly with flow and establish continuous junctions. In contrast, cells on fibronectin exhibit random orientation and disrupted barrier function. These differences arise from altered integrin signaling through divergent matrix compositions.

These platforms integrate three-dimensional matrices with controlled flow to enable systematic investigation of hemodynamic and biochemical variable interactions. Patient-specific geometries and human cells provide translational relevance while small scale enables parallel fabrication for higher throughput. However, complexity in fabrication and maintaining sterile perfusion presents practical challenges. Despite these limitations, organ-on-chip platforms provide valuable capabilities for investigating mechanisms under conditions closely approximating in vivo environments.

## 3. Gel-Based Therapeutic Strategies for Atherosclerosis

**Figure 5:**
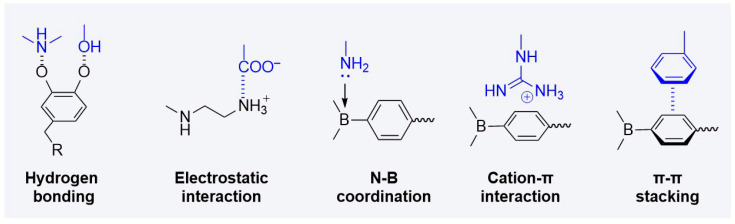
Fundamental molecular interactions in gel-based therapeutic systems.

Therapeutic gel platforms for atherosclerosis employ diverse chemical strategies to achieve controlled drug release, tissue adhesion, and stimuli-responsive behavior. These functional capabilities emerge from orchestrated molecular interactions within polymer networks. The design of effective gel systems requires an understanding of how basic intermolecular forces contribute to macroscopic material properties and therapeutic performance. Hydrogen bonding provides structural stability and enables reversible associations. Electrostatic interactions facilitate incorporation of charged therapeutics and modulate network crosslinking density. Coordination bonds through elements such as boron enable the formation of dynamic networks with tunable properties. Cation-π interactions and π-π stacking contribute to structural organization and molecular recognition. Figure 5 illustrates these fundamental molecular interactions underlying gel-based therapeutic systems. Natural polymer hydrogels, synthetic constructs, composite materials, and nanoscale carriers all leverage these interactions in different combinations to achieve specific therapeutic functions.

### 3.1. Macroscopic Gels for Localized Treatment

#### 3.1.1. Natural Polymer-Based Hydrogels

Natural polymer hydrogels derived from polysaccharides and proteins provide inherent biocompatibility and biodegradability for localized atherosclerosis treatment. These materials structurally resemble native extracellular matrix components, facilitating tissue integration while minimizing foreign body responses. Carboxyl groups in polysaccharides like hyaluronic acid and alginate enable covalent modification and crosslinking, whereas amine groups in chitosan permit electrostatic drug complexation [43]. Degradation products typically integrate into normal metabolic pathways, avoiding long-term accumulation concerns particularly important for vascular applications involving prolonged blood and tissue contact. Additionally, certain natural polymers possess intrinsic biological activities including anti-inflammatory and immunomodulatory properties that may contribute to therapeutic effects independently of or synergistically with loaded pharmaceutical agents.

**Figure 6:**
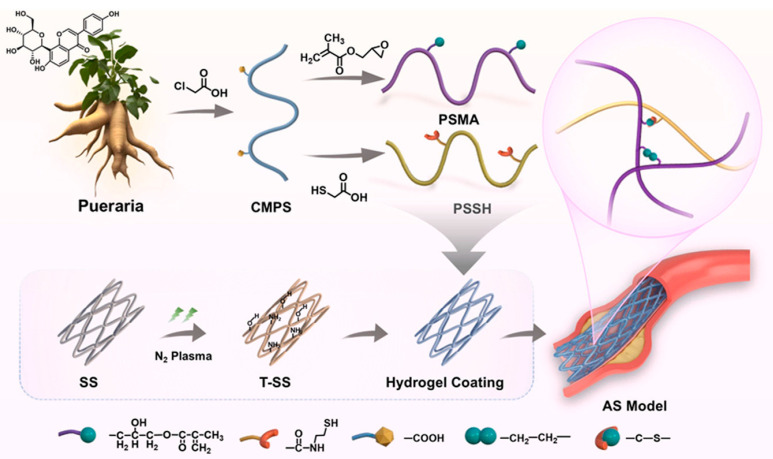
Plasma-reinforced dual-crosslinked Pueraria hydrogel coating for synergistic atherosclerosis intervention. Reproduced with permission from reference [47]. Open access.

Polysaccharide-based hydrogels demonstrate versatile applications through diverse crosslinking and stimuli-responsive strategies. For example, hyaluronic acid modified with catechol groups enables strong wet tissue adhesion for stent coatings, while disulfide crosslinking provides redox-responsive degradation [44]. When combined with allicin as a hydrogen sulfide donor, these coatings respond to oxidative stress in atherosclerotic lesions through controlled drug release. Testing in rabbit models confirms reduced restenosis through modulation of inflammatory pathways. Studies show that hyaluronic acid combined with cross-linkable block copolymers enables mechanical responsiveness [45]. These hydrogels exhibit differential drug release under varying stenosis degrees. The CD44 receptor-binding capacity of hyaluronic acid also enables macrophage targeting in composite formulations with alginate. Calcium-mediated ionic crosslinking of hyaluronic acid and alginate creates dual-network hydrogels suitable for adventitial injection. Loading with interleukin-33 antibody and injecting into rat aorta adventitia reduces neointimal thickness for three weeks following single administration [46]. Recent work on alginate-based formulations leveraging guluronic acid block coordination with calcium ions has provided tunable mechanical properties for stent coatings [47]. Incorporating polysaccharides derived from Pueraria lobata with dual crosslinking strategies shows superior prevention of restenosis and thrombosis (Figure 6). Chitosan’s cationic nature at physiological pH facilitates mucoadhesive delivery approaches that bypass hepatic first-pass metabolism. Chitosan nanoparticles incorporated into thermosensitive poloxamer matrices show sustained drug release with improved bioavailability. Nasal administration formulations show substantial bioavailability improvement compared to oral delivery in atherosclerosis models [48].

Peptide-based and protein-derived hydrogels leverage molecular recognition through designed amino acid sequences. Short peptides incorporating insulin-like growth factor-1 motifs conjugated with naproxen form self-assembling β-sheet nanofibers with dual functionality. Subcutaneous injection in atherosclerotic mice shows efficacy comparable to recombinant protein in reducing lesion area [49]. D-amino acid peptides with naphthalene groups achieve macrophage-selective targeting. These supramolecular hydrogels gradually disassemble after injection, enabling selective activation of reverse cholesterol transport [50]. Autologous platelet-rich gel represents a clinically accessible approach harnessing thrombin-mediated fibrin polymerization. The fibrin matrix enriched with platelet-derived growth factors provides angiogenic and endothelial function enhancement. Retrospective analysis of diabetic patients with lower limb atherosclerotic disease shows seventy percent pain reduction and substantial improvements in walking distance and ankle-brachial index after twelve weeks of treatment [51].

Despite these advantages, natural polymer platforms face inherent limitations that complicate their application in atherosclerosis interventions. Biological sourcing introduces batch-to-batch variability in molecular weight distributions and functional group densities. This inconsistency directly affects gelation kinetics and drug release profiles, creating challenges for drug-eluting stent coatings where reproducible performance is essential for regulatory approval and clinical safety [52]. Polysaccharide materials exhibit compositional fluctuations between production lots. Alginate guluronic acid content varies with cultivation conditions, causing unpredictable calcium-mediated crosslinking densities and degradation rates in adventitial depot applications [53]. Protein-derived materials present additional concerns in atherosclerosis treatment, where the disease itself involves chronic vascular inflammation. Xenogeneic collagens may elicit antibody responses that exacerbate existing inflammatory processes within plaques. Recombinant protein systems can retain residual endotoxins that trigger additional immune activation in already inflamed arterial tissue [54]. Polysaccharide matrices demonstrate high water retention, causing substantial swelling under physiological conditions. This volume expansion becomes problematic in space-constrained vascular applications, where excessive swelling could narrow lumens or compress surrounding tissue. Rapid enzymatic degradation limits sustained therapeutic delivery [55]. Atherosclerosis progression occurs over months to years, yet many natural polymer hydrogels degrade within weeks under proteolytic conditions present in inflamed plaques [56]. This temporal mismatch necessitates frequent reintervention or dosing adjustments. These constraints motivate development of synthetic polymer alternatives and hybrid composite formulations that balance biological recognition with manufacturing consistency and extended functionality.

#### 3.1.2. Synthetic Polymer-Based Hydrogels

**Figure 7:**
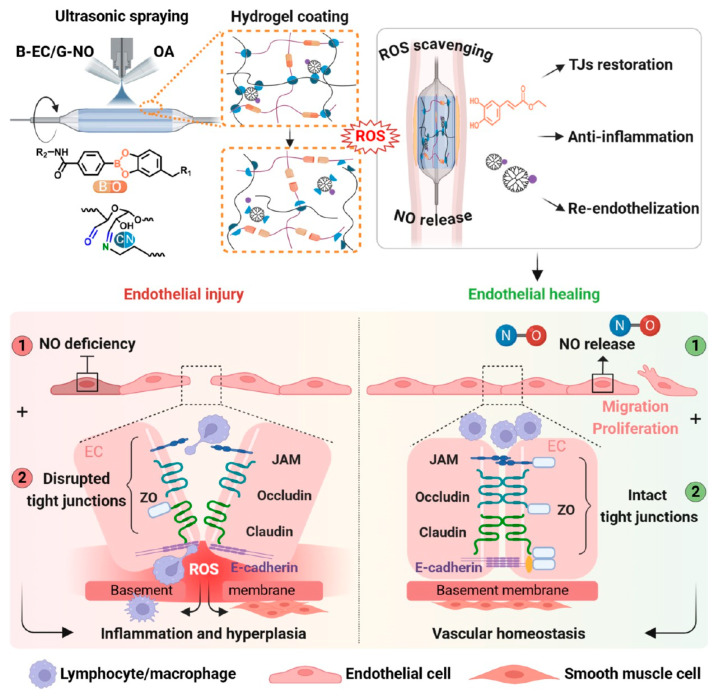
Sprayable, reactive oxygen species-responsive hydrogel coatings restore endothelial barrier integrity for functional vascular healing. Reproduced with permission from reference [60]. Copyright 2025, American Chemical Society.

Synthetic polymer hydrogels provide precise molecular control unavailable with biologically sourced materials. Defined molecular weights, narrow polydispersity, and specific functional group distributions enable systematic optimization of mechanical properties and degradation kinetics through rational design [57]. Polyethylene glycol, polyvinyl alcohol, and acrylic polymers represent widely investigated platforms for vascular applications [58]. The absence of batch-to-batch variability facilitates regulatory approval and clinical translation. Chemical versatility permits incorporation of stimuli-responsive moieties responding to oxidative stress, pH changes, or enzymatic activity characteristic of atherosclerotic microenvironments. Polyethylene glycol-based hydrogels have become prominent in vascular interventions due to biocompatibility and protein adsorption resistance. The antifouling properties arise from high chain mobility and strong hydration creating entropic barriers against protein deposition [59]. This characteristic proves valuable for drug-coated balloons, where minimizing unintended protein interactions maintains drug bioavailability during brief vessel contact. For example, dopamine-modified oxidized dextran crosslinked with caffeic acid derivatives and macromolecular nitric oxide donors through Schiff base chemistry creates dual responsive networks [60]. Phenylboronic ester linkages respond to elevated reactive oxygen species through hydrolytic cleavage, while Schiff bases provide pH-sensitive degradation. This architecture enables gradual disintegration at lesion sites characterized by oxidative stress and acidic conditions. Released caffeic acid scavenges reactive oxygen species and restores tight junction integrity, while nitric oxide delivery promotes endothelial proliferation. Application as sprayable balloon coatings shows effective barrier function restoration with reduced inflammation and smooth muscle proliferation. Catechol-mediated adhesion between dopamine residues and tissue surfaces enables drug retention despite transient balloon contact (Figure 7). Hydrogel-coated balloons enable localized delivery of molsidomine [61]. This nitric oxide donor accumulates in vessel walls with prolonged residence, undergoing gradual metabolic conversion. Studies in porcine models show restored wall pulsatility and thromboresistance. Long-term follow-up shows inhibition of restenotic lesion development.

Block copolymer hydrogels incorporating stimuli-responsive segments provide morphological transitions responding to environmental changes. Polyethylene glycol–block–polypropylene sulfide architecture exploits oxidation-sensitivity for controlled drug release [62]. This amphiphilic copolymer undergoes morphological transitions in response to oxidative conditions. This oxidation-triggered disassembly provides controlled drug release responsive to oxidative stress in atherosclerotic lesions. Loading 1,25-dihydroxyvitamin D3 into filomicelles enables stable encapsulation of this lipophilic immunomodulatory agent inhibiting nuclear factor kappa B signaling. Crosslinking drug-loaded filomicelles with multi-arm polyethylene glycol creates gel networks, where filaments serve as junctions. Following subcutaneous injection in atherosclerotic mice, these depots maintain high regulatory T cell levels in lymphoid organs and lesions for weeks through sustained low-dose delivery. Cryogenic electron microscopy confirms gradual filament oxidation and fragmentation into smaller micelles that exit the depot. The oxidation-responsive degradation couples material breakdown to pathological severity, automatically adjusting release kinetics.

**Figure 8:**
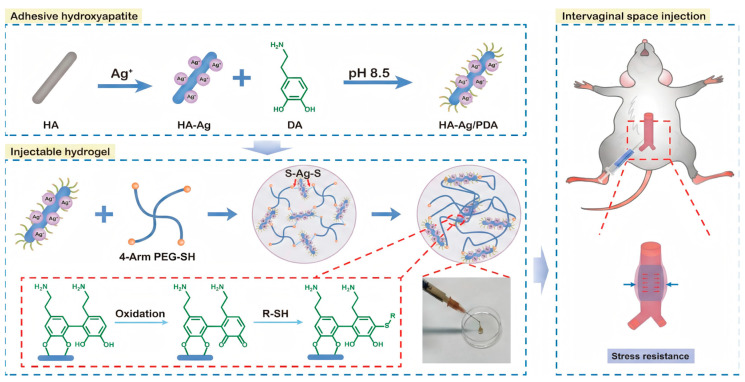
Injectable perivascular hydrogel for mechanical reinforcement of damaged arteries. Reproduced with permission from reference [63]. Copyright 2023, American Chemical Society.

Injectable hydrogels for perivascular delivery require mechanical properties withstanding hemodynamic forces while maintaining adventitial contact. A representative example demonstrates that thiol-terminated four-arm polyethylene glycol crosslinked with silver-doped nanohydroxyapatite modified with dopamine achieves high mechanical strength in injectable formulations [63]. Dopamine provides catechol groups for tissue adhesion and enables coordination with silver ions and calcium phosphate surfaces. When injected through perivascular interstitial space around damaged rat aortas, this hydrogel wraps around adventitia and significantly increases stress resistance. Tensile testing of explanted vessels demonstrates substantially higher pressure tolerance before rupture compared to controls. Biocompatibility assessment over three weeks reveals minimal immune infiltration, indicating well-tolerated silver-doped particles (Figure 8). The reinforcement mechanism relates to the hydrogel functioning as external scaffold redistributing mechanical loads across vessel walls.

These synthetic platforms demonstrate how chemical versatility achieves properties difficult to obtain with natural polymers. Multiple functional monomers within single chains enable simultaneous optimization of mechanics, degradation, and biological activity. Stimuli-responsive segments respond selectively to pathological signatures, while biocompatible spacers maintain cytocompatibility and resist nonspecific interactions. However, synthetic polymers typically lack intrinsic biological recognition elements, necessitating deliberate incorporation of bioactive ligands or reliance on passive physical properties. Selection between natural and synthetic approaches involves balancing biological sophistication against chemical control, with hybrid approaches increasingly employed to capture advantages of both material classes.

#### 3.1.3. Composite and Multifunctional Hydrogels

Composite hydrogels integrate natural and synthetic polymers or incorporate functional nanomaterials to overcome single-component limitations [64]. Natural polymers provide bioactivity and cell recognition but often exhibit insufficient mechanical strength. Synthetic polymers offer precise architectural control yet lack biological cues guiding tissue integration. Composite strategies harness complementary strengths through physical blending, covalent grafting, or interpenetrating network formation [65]. Functional nanoparticle incorporation extends capabilities by introducing catalytic activity, magnetic responsiveness, or imaging contrast unattainable through polymer chemistry alone [66,67].

Thermosensitive blends combining synthetic block copolymers with natural polysaccharides generate useful synergies. Poloxamer 407 undergoes reverse thermal gelation where aqueous solutions transition from low-viscosity liquids to gels at body temperature through micellization [68]. However, poloxamer gels exhibit limited mechanical strength and rapid dissolution. Incorporating sodium alginate addresses these limitations through calcium-mediated ionic crosslinking. Poloxamer combined with alginate enables endoluminal gel paving following coronary angioplasty [69]. Extending this concept, ternary blends incorporating hydroxymethyl cellulose have been developed for transcatheter arterial chemoembolization [70]. Hydroxymethyl cellulose increases liquid-state viscosity for catheter delivery while reinforcing gel networks through hydrogen bonding. When applied for embolization, this thermosensitive hydrogel achieves effective vessel occlusion. Temperature-triggered solidification enables controlled gelation after delivery. Poloxamer-alginate formulations can target atherosclerotic plaque neovascularization through local growth factor delivery [71]. Ultrasound-guided injection enables gradual release. Studies show effective reduction in plaque burden and neovascularization density.

**Figure 9:**
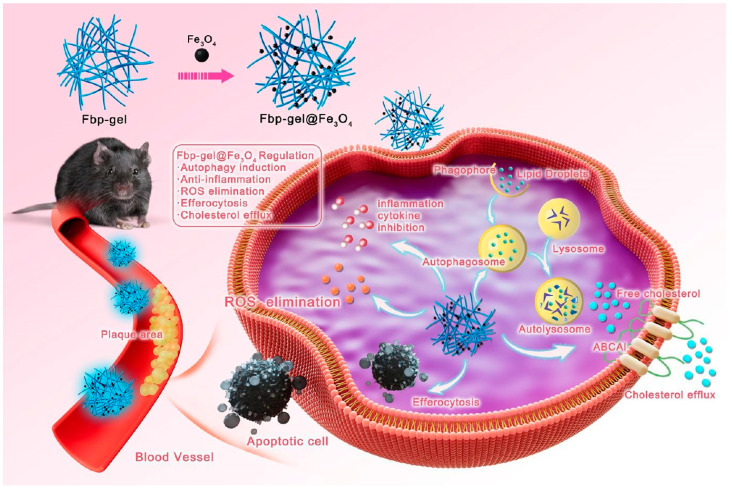
Functional peptide hydrogel for the synergistic treatment of atherosclerosis based on macrophage autophagy induction and anti-inflammation. Reproduced with permission from reference [73]. Copyright 2024, American Chemical Society.

Nanoparticle-incorporated hydrogels provide functional enhancements beyond organic polymers alone. Collagen hydrogels loaded with tannic acid-coated manganese-cobalt oxide nanoenzymes show promising applications for myocardial infarction secondary to coronary atherosclerosis [72]. The nanoenzymes provide catalytic antioxidant activity, while tannic acid coating offers additional chemical antioxidant capacity, and the collagen matrix modulates inflammatory cytokine profiles. These complementary mechanisms produce synergistic therapeutic effects exceeding individual components. The collagen component modulates inflammatory cytokine profiles. When injected into infarcted hearts, this composite shows synergistic therapeutic effects improving ventricular remodeling and cardiac function. Another composite strategy focuses on autophagy induction in atherosclerotic macrophages using iron oxide nanoparticles combined with naproxen-conjugated peptide hydrogels [73]. The self-assembling peptide provides anti-inflammatory action, while iron oxide activates autophagy through lysosomal accumulation. This autophagic response enhances macrophage cholesterol efflux while reducing inflammatory cytokine production and apoptosis, favoring plaque stability (Figure 9).

**Figure 10:**
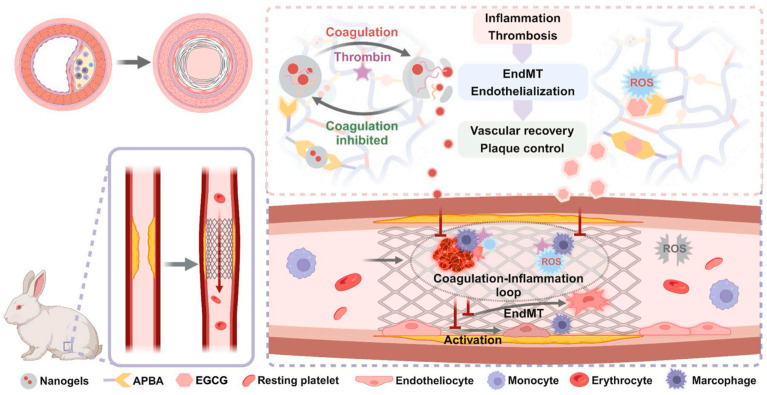
Dual-responsive nanogel-containing stent coating addressing coagulation–inflammation feedback. Reproduced with permission from reference [76]. Copyright 2025, Elsevier.

Liposome-incorporated and multi-responsive hydrogels represent advanced composite architectures. For instance, polyethylene glycol-norbornene crosslinked with thiolated heparin and supplemented with polyvinyl butyral forms networks designed for intimal injury repair following carotid endarterectomy [74]. Polyvinyl butyral reduces swelling and enhances wet surface adhesion. Cationic liposomes loaded with rapamycin are physically entrapped during gelation. Thiol-norbornene click chemistry enables rapid gelation through bioorthogonal mechanisms proceeding efficiently in blood without external catalysts. When spray-applied to injured arteries, this composite adheres to wet luminal surfaces and maintains stability under blood flow. Alternative approaches for sustained drug delivery have employed hyaluronic acid and sodium alginate hydrogels loaded with poly (lactic-co-glycolic acid) rapamycin nanoparticles for adventitial injection [75]. This dual-network structure maintains therapeutic drug levels in arterial walls for weeks following single administration. Treated vessels show significantly reduced neointimal thickness along with decreased inflammation. Recent advances in dual-responsive stent coatings integrate thrombin-responsive nanogels with antioxidant crosslinkers, exemplifying sophisticated control strategies [76]. Nanogels containing thrombin-cleavable peptide sequences release encapsulated apixaban during coagulation activation. Epigallocatechin gallate functions as crosslinker and antioxidant, detaching during oxidative stress. This dual architecture addresses coagulation-inflammation feedback loops contributing to stent thrombosis. Testing in atherosclerotic rabbits demonstrates inhibition of plaque deterioration through coordinated regulation of both pathways (Figure 10).

These composite and multifunctional hydrogels demonstrate how strategic complexity addresses multifaceted atherosclerosis pathophysiology. Natural-synthetic combinations balance biological recognition with mechanical robustness. Functional nanoparticle incorporation introduces catalytic and optical properties. Multi-responsive designs couple material behavior to multiple pathological signatures. Table 1 summarizes representative macroscopic hydrogel formulations discussed in this section, highlighting the diverse crosslinking strategies, application routes, and functional properties that enable localized atherosclerosis treatment. However, increased complexity introduces manufacturing reproducibility challenges and regulatory pathway uncertainties. Each additional component requires independent characterization, while interactions between components must be understood for predicting long-term performance.

While direct clinical applications of gel-based therapies for atherosclerotic plaques remain limited, related cardiovascular applications have entered clinical testing, providing important safety and feasibility data. Several hydrogel products have progressed to clinical trials for treating myocardial infarction and heart failure, conditions that represent severe complications of atherosclerosis. Two alginate-based hydrogels have completed early phase clinical evaluation. Algisyl-LVR, administered via epicardial injection in the AUGMENT-HF Phase II trial, showed improvements in cardiac function in patients with advanced heart failure without introducing significant safety concerns [77]. A larger follow-up trial is currently underway. IK-5001, delivered through intracoronary catheter injection in the RESERVATION-1 study, forms a gel matrix in the calcium-rich infarct environment [78]. Initial results indicated acceptable safety and improved exercise tolerance in myocardial infarction patients. An improved version of alginate hydrogel has also undergone clinical testing in China (NCT04781660), showing preliminary cardiac function improvements in heart failure patients. VentriGel, a decellularized porcine cardiac extracellular matrix hydrogel, completed Phase I clinical evaluation in the United States with 15 post-infarction patients receiving endocardial injections [79]. Six-month follow-up data supported safety and feasibility, leading to FDA approval for Phase II trials. These cardiovascular applications suggest that hydrogel-based interventions can be safely translated to human use, though direct targeting of atherosclerotic plaques requires further clinical investigation.

### 3.2. Nano- and Microgels for Systemic Delivery

#### 3.2.1. Polymer-Based Nanogels

Nanogels are crosslinked polymer networks with dimensions typically between 50 and 200 nanometers, enabling intravenous administration and potential accumulation at atherosclerotic sites [80]. Unlike macroscopic hydrogels functioning as localized depots, nanogels must navigate systemic circulation including clearance mechanisms and vascular extravasation. The three-dimensional crosslinked structure provides high drug-loading capacity and protects labile therapeutics within the polymer matrix. Surface modification enables incorporation of targeting ligands or stealth coatings [81]. However, the nanoscale design introduces distinct challenges related to colloidal stability, avoidance of rapid renal clearance, and achievement of sufficient circulation times for disease site accumulation. Size distribution requires careful control since polydispersity leads to heterogeneous pharmacokinetics and unpredictable clearance patterns. Surface charge also requires optimization, as highly charged particles activate complement and undergo rapid clearance, whereas near-neutral particles exhibit extended circulation [82].

**Figure 11:**
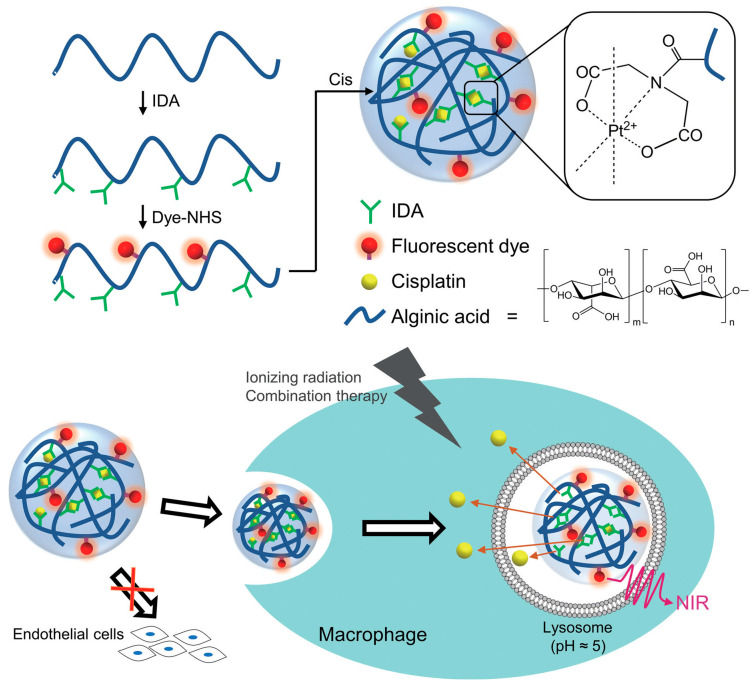
pH-responsive alginate-cisplatin nanogels for macrophage-targeted imaging and chemotherapy. Reproduced with permission from reference [85]. Copyright AME Publishing Company.

Natural polymer nanogels leverage polysaccharide biocompatibility while incorporating stabilizing crosslinks. For example, chitosan nanogels prepared through ionic gelation with tripolyphosphate demonstrate utility for oral delivery of lipid-lowering drugs [83]. Cationic nature enables electrostatic drug complexation and mucoadhesive properties prolonging gastrointestinal residence. Pravastatin-loaded chitosan nanogels show high entrapment efficiency and sustained release properties. Studies in hyperlipidemic rats reveal significant triglyceride and cholesterol reductions compared to commercial tablets, indicating enhanced bioavailability. Another approach employs interpenetrating network nanogels combining β-cyclodextrin with polyvinyl alcohol to address poor drug solubility through dual-network structures [84]. β-cyclodextrin cavities provide hydrophobic pockets for drug inclusion while polyvinyl alcohol contributes mechanical integrity. Rosuvastatin-loaded formulations show substantial solubility increases and rapid release. Testing in high-fat diet rats shows significantly lower lipid levels with improved atherosclerotic indices. Alginate modified with iminodiacetic acid exhibits enhanced platinum chelation capacity. When cisplatin serves simultaneously as crosslinking agent and drug, the resulting nanogels exhibit narrow size distributions [85]. These formulations show pH-sensitive drug release with preferential release under acidic conditions. Studies using macrophage cell lines demonstrate preferential uptake compared to endothelial cells, enabling selective imaging and chemotherapy (Figure 11). Trehalose-modified nanogels address autophagy induction mechanisms [86]. Copolymerization strategies enable effective trehalose incorporation while maintaining colloidal stability. Cell culture and animal studies show autophagy stimulation and significant plaque reduction.

Synthetic polymer nanogels provide compositional precision through controlled polymerization. Reversible addition-fragmentation chain transfer polymerization enables well-defined structures with narrow molecular weight distributions. Nitroxide-based nanogels prepared through this approach exhibit core–shell architectures providing antioxidant activity [87]. These nanoparticles show excellent stability compared to native enzymes. The nanogels reduce foam cell formation by protecting low-density lipoproteins from oxidation for up to one month. Polyethylene glycol nanogels enable protein encapsulation [88]. Using matrix metalloproteinase-responsive crosslinkers renders nanogels degradable in protease-rich plaque environments. Enzyme-loaded formulations show controlled release and reduced foam cell formation.

Stimuli-responsive nanogels responding to pathological signatures provide selective therapeutic activation. Fibronectin-based nanogels incorporating phenylboronic ester linkages exhibit pH-sensitive degradation combined with plaque targeting [89]. Phenylboronic ester crosslinks undergo accelerated hydrolysis under acidic conditions, enabling release proportional to local acidification. Drug loading enables targeted delivery with minimal premature leakage. Combination with ultrasound-targeted microbubble destruction enhances therapeutic efficacy. Studies in atherosclerotic rabbits demonstrate superior plaque suppression compared to non-targeted formulations. Mechanical stress-responsive nanogels provide an alternative triggering mechanism exploiting altered hemodynamics in stenotic vessels [90]. Physically crosslinked networks exhibit tunable disintegration upon elevated shear stress exposure. These nanogels remain intact under normal flow but release cargo in stenotic regions with elevated shear stress. When loaded with anticoagulants and tested in flow systems, these nanogels exhibit differential release responding to local hemodynamic conditions.

These nanogel platforms show how polymer chemistry tailors nanocarrier properties to atherosclerosis treatment challenges. Natural polymer nanogels leverage biocompatibility and biodegradability, while synthetic approaches provide compositional control. When interpreting therapeutic outcomes, the intrinsic biological activities of polymer matrices should be considered alongside loaded drug effects, as many polysaccharides and certain synthetic polymers exhibit anti-inflammatory or immunomodulatory properties. Stimuli-responsive designs couple drug release to pathological signatures including acidic pH, oxidative stress, or altered hemodynamics. Representative examples of polymer-based and inorganic–organic composite nanogels for systemic atherosclerosis therapy are compiled in Table 2, illustrating the progression from conventional drug carriers to theranostic platforms integrating imaging capabilities. However, all designs must address colloidal stability, protein corona formation, and balancing circulation time against disease site accumulation. Surface modification with polyethylene glycol or zwitterionic polymers extends circulation by reducing opsonization, while targeting ligands enable active plaque marker recognition.

#### 3.2.2. Inorganic–Organic Composite Nanogels

Inorganic–organic composite nanogels integrate paramagnetic ions or metal complexes within polymer networks to combine therapeutic delivery with diagnostic imaging functionality. This theranostic integration addresses challenges in therapeutic efficacy variation among individuals [93]. Incorporating imaging contrast agents enables monitoring of nanogel accumulation, drug release, and therapeutic responses. This approach may provide opportunities for identifying non-responding patients, adjusting dosing regimens, and stratifying patients based on imaging profiles [94]. Gadolinium chelates are commonly employed for magnetic resonance imaging due to strong T1 contrast effects, while paramagnetic nitroxide radicals offer both antioxidant activity and contrast. Design challenges involve maintaining colloidal stability of inorganic components, ensuring metal ions remain securely chelated to prevent toxic release, and achieving sufficient contrast agent loading without compromising drug delivery performance or circulation properties [95].

**Figure 12:**
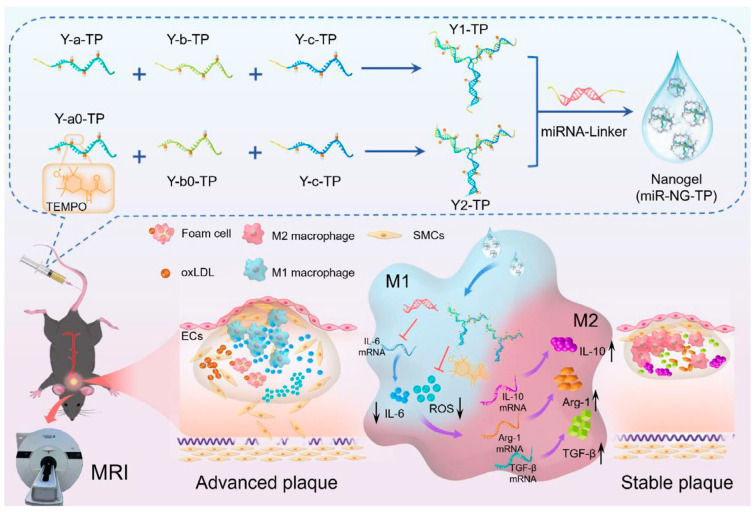
DNA-TEMPO nanogels combining antioxidant activity with magnetic resonance imaging capability. Reproduced with permission from reference [91]. Copyright 2025, John Wiley and Sons.

**Figure 13:**
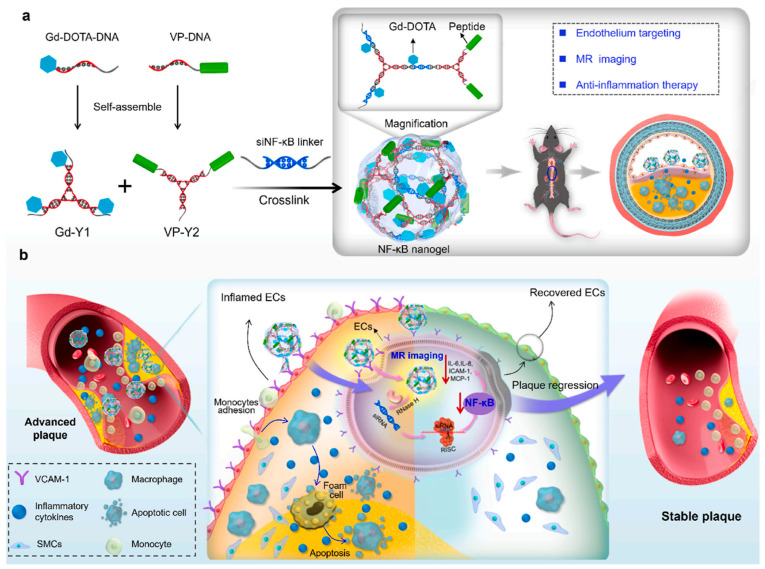
VCAM-1-targeted DNA-Gd-DOTA nanogels for theranostic atherosclerosis treatment. Reproduced with permission from reference [92]. Copyright 2025, Elsevier.

DNA-based nanogels incorporating paramagnetic functionality combine nucleic acid self-assembly with integrated imaging capability [96]. These structures provide both magnetic resonance contrast and treatment delivery. Nitroxide-grafted DNA nanogels crosslinked with therapeutic microRNA combine reactive oxygen species scavenging with imaging capacity [91]. Studies in atherosclerotic mice show plaque accumulation and therapeutic effects (Figure 12). Gadolinium–DOTA complexes conjugated to DNA strands achieve stronger contrast for clinical imaging [92]. By incorporating VCAM-1-targeting peptides, these nanogels show endothelial targeting and anti-inflammatory effects. Individual imaging studies suggest that early contrast enhancement may predict therapeutic responses (Figure 13).

These composite nanogels incorporating paramagnetic contrast agents show potential for personalized atherosclerosis treatment guided by imaging biomarkers. However, clinical translation of these theranostic nanogels remains limited. Unlike macroscopic hydrogels where several formulations have reached clinical testing for cardiovascular applications, nanogel platforms for atherosclerosis remain primarily in preclinical development stages, reflecting the additional complexities of systemic delivery including circulation stability, targeting efficiency, and long-term biodistribution concerns.

### 3.3. Comparative Analysis of Gel Platforms for Atherosclerosis Treatment

Macroscopic hydrogels and nanoscale formulations operate through fundamentally different pharmacokinetic principles [97]. Localized depots maintain drug concentrations at implantation sites through sustained release over weeks to months. Their efficacy depends on material retention against arterial pulsatile flow and controlled degradation matching vascular healing timescales. Therapeutic effects remain spatially confined within several millimeters from gel surfaces through diffusion-limited transport. This localization enables high concentrations at treatment sites while minimizing systemic exposure. Systemically administered nanogels face opposing challenges. Particle size critically determines clearance routes where formulations below ten nanometers undergo rapid renal filtration, while those exceeding two hundred nanometers experience accelerated hepatosplenic uptake [98]. The therapeutic window between fifty and one hundred fifty nanometers provides extended circulation but typically achieves only modest plaque enrichment of two to five-fold compared to blood levels. This limited targeting efficiency necessitates repeated dosing [99]. Extravasation through inflamed endothelium proceeds slowly and size-selectively. These divergent pharmacokinetic behaviors establish distinct optimization priorities where macroscopic gels require mechanical robustness, while nanogels demand colloidal stability and prolonged circulation.

Material composition involves tradeoffs between manufacturing precision and biological functionality. Natural polysaccharides exhibit inherent batch-to-batch compositional variability from biological sourcing. Molecular weight distributions and functional group densities fluctuate between production batches, causing inconsistent gelation kinetics and degradation rates [100]. Protein-derived materials additionally face immunogenicity concerns, where xenogeneic collagens can elicit antibody responses in sensitive populations [101]. Synthetic polymers provide superior manufacturing reproducibility through controlled polymerization techniques. Batch consistency and molecular weight precision facilitate regulatory characterization and large-scale production under good manufacturing practice conditions [102]. However, synthetic backbones lack intrinsic cell recognition motifs. Achieving biological responsiveness requires deliberate incorporation of cleavable linkages or peptide sequences. Stimuli-responsive mechanisms face kinetic limitations, where response rates must balance sensitivity against premature activation during storage or circulation. Composite strategies combining natural and synthetic components capture complementary advantages but increase formulation complexity. Manufacturing scalability generally favors synthetic platforms, while regulatory pathways for natural polymers benefit from historical precedent despite requiring extensive source characterization.

Application-specific requirements impose distinct performance criteria that guide material selection. Stent coatings constrain layer thickness to maintain device flexibility during deployment [103]. This geometric limitation restricts drug-loading capacity and necessitates erosion-controlled release mechanisms rather than diffusion-based systems. Coating adhesion must withstand expansion-induced mechanical strains. Adventitial injection tolerates larger volumes but requires adequate gel stiffness to resist displacement around mobile vessels [104]. Natural polymer matrices often achieve necessary mechanical properties only at higher concentrations that may compress surrounding tissue. Drug-coated balloons demand unique rheological properties combining low viscosity during catheter delivery with rapid tissue adhesion during brief inflation periods. Formulations must enable substantial drug transfer into arterial walls despite luminal washout [105]. Systemic delivery platforms face fundamentally different challenges, where achieving therapeutic plaque concentrations from dilute circulating levels requires either active targeting through receptor recognition or passive accumulation through enhanced permeability. Ligand-mediated targeting provides modest improvements of two- to three-fold over non-targeted controls. Stimuli-responsive release triggered by plaque microenvironment signatures can improve selectivity but faces limitations from incomplete activation and premature leakage during circulation. These application-specific constraints necessitate matching material capabilities to procedural requirements and therapeutic objectives.

## 4. Challenges and Future Perspectives

### 4.1. Current Challenges and Limitations

Translating gel formulations from laboratory synthesis to clinical-scale manufacturing encounters substantial technical barriers. Crosslinking chemistries optimized in small batches often fail during scale-up, where reaction conditions become difficult to control uniformly [106]. Catechol oxidation reactions sensitive to dissolved oxygen concentration exhibit irreproducible gelation kinetics in large production vessels, where oxygen gradients develop despite mechanical stirring. Click chemistry approaches requiring precise stoichiometric ratios between reactive groups become challenging when mixing hundred-milliliter volumes. Incomplete homogenization creates spatial heterogeneity in crosslink density that manifests as inconsistent mechanical properties and drug release profiles across production lots. Sterility requirements impose additional constraints [107]. Injectable hydrogel formulations demand terminal sterilization or aseptic processing to meet pharmacopeial standards. Gamma irradiation damages stimuli-responsive linkages incorporated for controlled degradation. Phenylboronic ester bonds undergo partial hydrolysis during autoclaving. Disulfide crosslinks experience oxidative cleavage under radiation exposure. These chemical instabilities force adoption of aseptic manufacturing protocols that substantially increase production costs and contamination risks during multi-step synthesis procedures. Residual solvent removal requires extensive validation [108]. Organic solvents employed during polymer synthesis or drug loading must be reduced below the International Conference on Harmonization limits before clinical use. Dimethyl sulfoxide and tetrahydrofuran commonly used for hydrophobic drug solubilization necessitate prolonged vacuum drying. Incomplete removal leaves toxic residues that accumulate in arterial walls following repeated local administration in atherosclerosis treatment applications.

Macroscopic hydrogels applied as stent coatings or adventitial injections must withstand hemodynamic forces substantially exceeding stresses replicated in benchtop testing. Arterial pulsatile flow generates wall shear stresses between one and seven pascals in diseased vessels [109]. Cyclic strain during cardiac cycles subjects perivascular depots to repeated deformation. These mechanical demands frequently induce premature coating delamination or bulk gel fragmentation within days of implantation despite weeks of apparent stability during static in vitro culture. Achieving adequate wet tissue adhesion represents another persistent obstacle. Blood proteins rapidly adsorb onto exposed gel surfaces and interfere with catechol-mediated bonding mechanisms optimized for clean substrates. Fibrinogen and albumin accumulation within minutes of blood exposure creates protein barriers, preventing direct contact between adhesive functional groups and arterial tissue [110]. Current adhesive strategies perform unpredictably when applied through blood-filled catheters onto protein-coated vessel walls. Degradation kinetics must align with vascular healing timescales, as premature disintegration causes subtherapeutic drug exposure, while excessive persistence beyond three months triggers chronic foreign body responses. However, predicting long-term degradation behavior from accelerated in vitro testing remains unreliable. Hydrolytic cleavage rates measured at elevated temperature or pH poorly correlate with enzymatic degradation occurring within protease-rich atherosclerotic plaques over months.

Atherosclerotic plaques exhibit complex structural heterogeneity that complicates therapeutic delivery. Regions vary from lipid accumulations with minimal extracellular matrix to densely collagenous caps and mineralized deposits [111]. Each region presents different diffusion barriers and enzymatic activities influencing local drug release kinetics. Lipid accumulations impede hydrophilic drug transport through hydrophobic barriers. Calcified regions create physical obstacles preventing depot penetration. Dense collagen networks restrict macromolecular diffusion from gel surfaces into underlying tissue. Individual patients exhibit substantial variability in inflammatory marker expression, oxidative stress levels, and protease activity profiles. These biochemical differences cause stimuli-responsive gels designed to activate under specific thresholds to perform inconsistently across populations [112]. Formulations releasing drugs upon exposure to matrix metalloproteinases exhibit wide variations in release rates between patients with different protease expression patterns. Oxidation-responsive systems activated by reactive oxygen species show unpredictable performance in patients with varying degrees of oxidative stress within lesions. This biological variability complicates clinical trial design, where treatment responses vary substantially among individuals receiving identical formulations.

Long-term safety assessment remains inadequate for many formulations, as accelerated degradation studies lasting weeks cannot reliably predict tissue responses over months to years that gel remnants may persist at implantation sites. Degradation products from synthetic polymers accumulate in surrounding arterial tissue with uncertain long-term consequences. Oligomeric fragments below renal filtration thresholds distribute systemically and may undergo hepatic metabolism through pathways not fully characterized. Composite hydrogels incorporating metallic or ceramic nanoparticles raise additional concerns. Functional nanoparticles added for catalytic antioxidant activity or imaging contrast undergo slow dissolution releasing ions over extended periods [113]. Manganese and iron oxide particles incorporated in several formulations persist in vessel walls beyond intended treatment duration. Gadolinium-containing nanogels designed for magnetic resonance imaging face scrutiny following recognition of tissue deposition after repeated contrast agent administration. Similar accumulation patterns may occur with therapeutic gel formulations, where nanoparticle clearance from arterial walls proceeds slowly [114]. The inflammatory microenvironment within atherosclerotic plaques potentially alters nanoparticle dissolution kinetics compared to healthy tissue. Acidic pH and elevated protease activity could accelerate particle degradation and ion release beyond rates observed during standard biocompatibility testing in normal tissues.

Regulatory classification for gel-based atherosclerosis therapies remains incompletely defined across major jurisdictions. Macroscopic hydrogels applied as stent coatings may be evaluated as device constituent parts subject to biocompatibility testing under ISO 10993 standards [115]. Injectable depot formulations delivering pharmaceuticals constitute combination products requiring separate assessment of material and drug components. Systemically administered nanogels typically follow drug regulatory pathways despite containing non-biodegradable polymeric structures. This classification ambiguity complicates development strategies, where regulatory requirements differ substantially between pathways. Drug-device combinations face particular scrutiny regarding component interactions and combined risk profiles that exceed individual component risks. Coating detachment releasing gel fragments into circulation creates embolic hazards not present with either stents or drugs independently [116]. Clinical implementation faces practical constraints in procedural compatibility. Adventitial injection around coronary arteries requires precise volume control, where excessive volumes compress vessels or leak into surrounding mediastinal tissue. Insufficient volumes fail to establish therapeutic concentration gradients. Precise volume delivery at the narrow therapeutic range demands specialized catheter designs enabling accurate delivery through tortuous vascular access routes. Formulation viscosity must balance injectability through small-gauge needles against rapid gelation preventing post-injection dispersion. This rheological window narrows considerably when incorporating particulate drug carriers or achieving high loading densities. Drug-coated balloon applications face analogous constraints where coating adhesion during brief inflation periods competes with drug transfer efficiency into arterial walls despite luminal washout.

### 4.2. Future Directions and Opportunities

Computational approaches integrating artificial intelligence and machine learning offer substantial potential for accelerating gel formulation optimization beyond current trial-and-error methodologies [117]. Training algorithms on datasets correlating polymer composition with mechanical properties, degradation kinetics, and drug release profiles could enable rapid computational screening of candidate formulations before synthesis. High-throughput platforms combining automated synthesis with parallel characterization would support development of robust predictive models guiding rational materials design. Multi-functional platform development continues advancing toward integrated systems combining therapeutic release with diagnostic monitoring capabilities. Future iterations may incorporate complementary imaging modalities, enabling comprehensive assessment of therapeutic responses, such as combining magnetic resonance contrast with optical reporters for multi-scale visualization spanning biodistribution to cellular uptake patterns.

Biomimetic design strategies leveraging cell membrane coatings present opportunities for improving nanocarrier performance through natural biological recognition mechanisms [118]. Red blood cell membrane coating extends circulation through presentation of self-markers inhibiting phagocytic clearance, while platelet membrane coating enables adhesion to disrupted endothelium. Applications extending toward primary prevention represent another direction, where long-acting gel formulations could enable proactive cardiovascular risk management in high-risk populations through sustained, multi-month release of protective agents. Such preventive platforms require extensive safety validation, given administration to asymptomatic individuals, but offer potential for population-level atherosclerosis burden reduction through early intervention before irreversible vascular remodeling occurs.

## 5. Conclusions

Gel-based technologies provide versatile platforms spanning from in vitro disease models to therapeutic delivery vehicles for atherosclerosis research and intervention. Hydrogel matrices enable investigation of plaque microenvironment effects on cellular behaviors under controlled three-dimensional conditions. Microfluidic implementations introduce physiological flow patterns that critically influence endothelial phenotype. Macroscopic hydrogels for localized treatment leverage multiple material strategies. Natural polymers provide biocompatibility and often possess intrinsic biological activities including anti-inflammatory and immunomodulatory properties. Synthetic polymers offer chemical versatility and manufacturing consistency. Composite designs integrate multiple material classes. These approaches achieve sustained drug release at specific vascular sites through stent coatings, adventitial injections, or catheter-delivered depots. Several hydrogel formulations have progressed to clinical testing for cardiovascular applications, demonstrating feasibility and safety in early phase trials. Nanogels enable systemic administration with stimuli-responsive drug release. Release triggers include pathological signatures such as oxidative stress, acidic pH, and altered hemodynamics in stenotic vessels. Composite nanogels incorporating paramagnetic contrast agents advance toward theranostic paradigms. These enable patient stratification and treatment monitoring through integrated imaging capabilities. Despite substantial progress, challenges remain in manufacturing consistency, long-term safety validation, and regulatory pathway definition. When interpreting therapeutic outcomes, the intrinsic biological activities of polymer matrices should be considered alongside loaded drug effects, as many polysaccharides and certain synthetic polymers exhibit anti-inflammatory or immunomodulatory properties. Ongoing developments in multi-functional integration, artificial intelligence-guided design, personalized formulations, and biomimetic approaches position gel technologies to substantially impact atherosclerosis management through improved spatial and temporal control over therapeutic interventions.

## Figures and Tables

**Table 1:** Macroscopic hydrogels for localized atherosclerosis treatment.

Material System	Crosslinking and Responsiveness	Application Route	Key Material Properties	Reference
Catechol-modified HA with cystamine	Amide bonding with disulfide bonds, redox-responsive to oxidative stress	stent coating	H_2_S release from allicin, intelligent inflammation response, endothelial repair promotion	[44]
HA/SA blend	Ionic gelation with CaCO_3_/Ca^2+^	adventitial injection	IL-33 antibody delivery, controlled degradation, neointimal hyperplasia inhibition	[46]
Methacrylated Pueraria polysaccharide	Dual-network, plasma-reinforced interface	Stent coating	Controlled flavonoid release, endothelial recovery, SMC phenotype modulation	[47]
Chitosan-based with glyceryl monooleate	pH-sensitive, nasal mucoadhesive	Nasal administration	Felodipine-loaded invasomes, 3.37-fold bioavailability increase, enhanced permeation	[48]
D-Nap-GFFY peptide nanofibers	Self-assembly into supramolecular nanofibers, scavenger receptor-mediated	Subcutaneous injection	IGF-1 mimetic with anti-inflammatory naproxen, macrophage-selective uptake, no hepatic lipogenesis	[49]
D-Nap-GFFY peptide with T0901317	Self-assembled peptide nanofibers	Subcutaneous injection	Macrophage LXR activation, ABCA1/G1 upregulation, Kupffer cell M2 polarization	[50]
Oxidized dextran with caffeate prodrug (B-EC) and G-NO donor	Dual-dynamic covalent, ROS-responsive	Ultrasonic spray on balloon	Self-healing, controlled antioxidant/NO release, tight junction restoration, wet adhesion	[60]
Hydrogel-coated angioplasty balloon	Stable coating with >72 h arterial retention	Balloon catheter delivery	Molsidomine delivery, slow NO release, vasodilation, anti-thrombotic and anti-proliferative	[61]
Pluronic F407/Alginate blend	Thermal gelation + ionic Ca^2+^ crosslinking	Catheter delivery for endoluminal gel paving	Temperature-responsive gelation, nucleic acid drug compatibility, soft gel formation	[69]
Poloxamer 407/SA	Thermal gelation + ionic Ca^2+^ crosslinking, self-healing	Ultrasound-guided abdominal aorta injection	PEDF delivery, VEGFA-dependent neovascularization inhibition, CD31 and MMP-2/9 modulation	[71]
Short peptide hydrogel with Fe_3_O_4_ and NSAID	Self-assembly with naproxen as crosslinker	Subcutaneous injection	Autophagy induction via Fe_3_O_4_, anti-inflammatory synergy, foam cell reduction, ROS suppression	[73]
PEG-norbornene with heparin and liposomes	Photoinitiated click chemistry, PVB-incorporated	Spray application for CEA repair	Ultra-fast wet adhesion, rapamycin-loaded liposomes, anti-coagulation, blood flow resistance	[74]
Nanogel-composite coating with EGCG	Thrombin-responsive apixaban release + ROS-responsive EGCG detachment	Stent coating	Self-adaptive dual-responsive, coagulation-inflammation loop regulation, EndMT inhibition	[76]

**Table 2:** Nanogels for systemic atherosclerosis therapy.

Nanogel Type	Size and Responsive Trigger	Therapeutic Cargo	Functional Integration	Reference
Chitosan nanoparticles converted to nanogel with poloxamer	Ionic gelation, sustained release	Pravastatin sodium	Oral delivery, high entrapment, reduced hemolytic toxicity, hyperlipidemia management	[83]
β-Cyclodextrin/PVA-co-AMPS IPN nanogels	Free radical polymerization with MBA crosslinking, rapid dissolution	Rosuvastatin	>70% entrapment efficiency, >90% release in 5–30 min, improved pharmacodynamic efficacy	[84]
Alginate modified with iminodiacetic acid, ATTO655-labeled	~100 nm, highly pH-responsive	Cisplatin	Macrophage-selective uptake, NIR fluorescence imaging, combined chemo/radiotherapy, reduced therapeutic dose	[85]
6-O-Acryloyl-trehalose copolymer nanogels	67 nm, ester bond cleavage for trehalose release, ~58% w/w conjugation	Trehalose	Autophagy stimulation in foam cells, lipid efflux enhancement, non-hemolytic, excellent colloidal stability in serum	[86]
RAFT-mediated nitroxide nanogels	30–40 nm, oxidation-responsive	Nitroxide radicals	SOD enzymatic mimicry, multi-ROS scavenging, LDL protection for 1 month, foam cell inhibition	[87]
PEG crosslinked with MMP-responsive elements	Confined aqueous droplet synthesis, MMP-triggered release	Paraoxonase-1 enzyme	Electrostatically driven template polymerization, ox-LDL reduction, macrophage foam cell prevention	[88]
Fibronectin-modified phenylboronic acid-PEG nanogels	pH-sensitive Schiff base linkages	Curcumin	RGD sequence-integrin targeting, UTMD-enhanced delivery, anti-inflammatory + antioxidant, plaque progression attenuation	[89]
Mechanosensitive PEG-based nanogels	Tunable disintegration upon stenotic shear stress levels, size-adjustable	Heparin	Stenosis-activated release, minimal passive leakage, similar clot lysis efficiency as free drug, hemocompatible	[90]
TEMPO-grafted Y-shaped DNA blocks crosslinked by miRNA	Paramagnetic TEMPO via phosphorothioate groups, ROS-scavenging	miR-146a-5p	MRI contrast, dynamic disease monitoring, oxidative stress relief, plaque regression and stabilization	[91]
Gd-DOTA and VCAM-1 peptide-conjugated Y-DNA assembled with siRNA	Nucleic acid self-assembly, inflamed endothelium-targeted	siNF-κB	MRI-based visualization, VCAM-1 targeting to inflammatory endothelium	[92]

## Data Availability

No new data were created or analyzed in this study.

## References

[1] Libby P., Buring J.E., Badimon L., Hansson G.K., Deanfield J., Bittencourt M.S., Tokgözoğlu L., Lewis E.F. (2019). Atherosclerosis. Nat. Rev. Dis. Primers.

[2] Fredman G. (2020). Devouring Atherosclerotic Plaques. Nat. Nanotechnol..

[3] Le Bras A. (2018). Chronotherapy for Atherosclerosis. Nat. Rev. Cardiol..

[4] Hetherington I., Totary-Jain H. (2022). Anti-Atherosclerotic Therapies: Milestones, Challenges, and Emerging Innovations. Mol. Ther..

[5] Andreou I., Stone P.H. (2016). In-Stent Atherosclerosis at a Crossroads: Neoatherosclerosis … or Paleoatherosclerosis?. Circulation.

[6] Joner M., Koppara T., Byrne R.A., Castellanos M.I., Lewerich J., Novotny J., Guagliumi G., Xhepa E., Adriaenssens T., Godschalk T.C. (2018). Neoatherosclerosis in Patients With Coronary Stent Thrombosis. JACC Cardiovasc. Interv..

[7] Liu X., Inda M.E., Lai Y., Lu T.K., Zhao X. (2022). Engineered Living Hydrogels. Adv. Mater..

[8] Kuzina M.A., Kartsev D.D., Stratonovich A.V., Levkin P.A. (2023). Organogels versus Hydrogels: Advantages, Challenges, and Applications. Adv. Funct. Mater..

[9] Sun F., Zhang Y., Zhang B., Qiao D., Xie F. (2025). Fibrous Protein Gels: Nanoscale Features Governing Gelation Behavior and Gel Properties. Adv. Colloid Interface Sci..

[10] Liu W., Gong X., Zhu Y., Wang J., Ngai T., Wu C. (2019). Probing Sol–Gel Matrices and Dynamics of Star PEG Hydrogels Near Overlap Concentration. Macromolecules.

[11] Gu X., Du L., Lin R., Ding Z., Guo Z., Wei J., Li Y. (2025). How Advanced Is Nanomedicine for Atherosclerosis?. Int. J. Nanomed..

[12] Chen J., Zhang X., Millican R., Lynd T., Gangasani M., Malhotra S., Sherwood J., Hwang P.T., Cho Y., Brott B.C. (2022). Recent Progress in in Vitro Models for Atherosclerosis Studies. Front. Cardiovasc. Med..

[13] Guan S., Li J., Zhang K., Li J. (2021). Environmentally Responsive Hydrogels for Repair of Cardiovascular Tissue. Heart Fail. Rev..

[14] Grimaudo M.A., Concheiro A., Alvarez-Lorenzo C. (2019). Nanogels for Regenerative Medicine. J. Control. Release.

[15] Lei C., Li Q., Chen W., Yu G. (2025). Biopolymeric Gels: Advancements in Sustainable Multifunctional Materials. Adv. Mater..

[16] Zhao L., Zhou Y., Zhang J., Liang H., Chen X., Tan H. (2023). Natural Polymer-Based Hydrogels: From Polymer to Biomedical Applications. Pharmaceutics.

[17] Zhang H., Gao X., Wang Y., Wu J., Zhang H., Jiang M., Wang W., Yan C. (2025). Natural Source Protein Hydrogels: Emerging Materials for Sports Injury Management and Protection. Int. J. Biol. Macromol..

[18] Ajoolabady A., Pratico D., Lin L., Mantzoros C.S., Bahijri S., Tuomilehto J., Ren J. (2024). Inflammation in Atherosclerosis: Pathophysiology and Mechanisms. Cell Death Dis..

[19] Theofilis P., Oikonomou E., Tsioufis K., Tousoulis D. (2023). The Role of Macrophages in Atherosclerosis: Pathophysiologic Mechanisms and Treatment Considerations. Int. J. Mol. Sci..

[20] Gurgoglione F.L., Denegri A., Russo M., Calvieri C., Benatti G., Niccoli G. (2023). Intracoronary Imaging of Coronary Atherosclerotic Plaque: From Assessment of Pathophysiological Mechanisms to Therapeutic Implication. Int. J. Mol. Sci..

[21] Vu L.T., Jain G., Veres B.D., Rajagopalan P. (2015). Cell Migration on Planar and Three-Dimensional Matrices: A Hydrogel-Based Perspective. Tissue Eng. Part B Rev..

[22] Jordan A.M., Kim S.-E., Van De Voorde K., Pokorski J.K., Korley L.T.J. (2017). In Situ Fabrication of Fiber Reinforced Three-Dimensional Hydrogel Tissue Engineering Scaffolds. ACS Biomater. Sci. Eng..

[23] Bronner-Shtrauchler O., Nativ-Roth E., Sanchez D.S., Zaiden M., Vidavsky N. (2024). Multimodal Characterization of the Collagen Hydrogel Structure and Properties in Response to Physiologically Relevant pH Fluctuations. Acta Biomater..

[24] Zuniga K., Ghousifam N., Shaffer L., Brocklehurst S., Van Dyke M., Christy R., Natesan S., Rylander M.N. (2024). Development of a Static Avascular and Dynamic Vascular Human Skin Equivalent Employing Collagen/Keratin Hydrogels. Int. J. Mol. Sci..

[25] Ruiz J.L., Hutcheson J.D., Cardoso L., Bakhshian Nik A., Condado De Abreu A., Pham T., Buffolo F., Busatto S., Federici S., Ridolfi A. (2021). Nanoanalytical Analysis of Bisphosphonate-Driven Alterations of Microcalcifications Using a 3D Hydrogel System and in Vivo Mouse Model. Proc. Natl. Acad. Sci. USA.

[26] Hutcheson J.D., Goettsch C., Bertazzo S., Maldonado N., Ruiz J.L., Goh W., Yabusaki K., Faits T., Bouten C., Franck G. (2016). Genesis and Growth of Extracellular-Vesicle-Derived Microcalcification in Atherosclerotic Plaques. Nat. Mater..

[27] Jia Y.-G., Jin J., Liu S., Ren L., Luo J., Zhu X.X. (2018). Self-Healing Hydrogels of Low Molecular Weight Poly(Vinyl Alcohol) Assembled by Host–Guest Recognition. Biomacromolecules.

[28] Mahamoud M.M., Ketema T.M., Kuwahara Y., Takafuji M. (2024). Enhancement of Mechanical Properties of Benign Polyvinyl Alcohol/Agar Hydrogel by Crosslinking Tannic Acid and Applying Multiple Freeze/Thaw Cycles. Gels.

[29] Poree J., Chayer B., Soulez G., Ohayon J., Cloutier G. (2017). Noninvasive Vascular Modulography Method for Imaging the Local Elasticity of Atherosclerotic Plaques: Simulation and In Vitro Vessel Phantom Study. IEEE Trans. Ultrason. Ferroelectr. Freq. Control..

[30] Chueh J.-Y., Van Der Marel K., Gounis M.J., LeMatty T., Brown T.R., Ansari S.A., Carroll T.J., Buck A.K., Zhou X.J., Chatterjee A.R. (2018). Development of a High Resolution MRI Intracranial Atherosclerosis Imaging Phantom. J. NeuroInterventional Surg..

[31] Razzi F., Lovrak M., Gruzdyte D., Den Hartog Y., Duncker D.J., Van Esch J.H., Van Steijn V., Van Beusekom H.M.M. (2022). An Implantable Artificial Atherosclerotic Plaque as a Novel Approach for Drug Transport Studies on Drug-Eluting Stents. Adv. Healthc. Mater..

[32] Skelton M.L., Gentry J.L., Astrab L.R., Goedert J.A., Earl E.B., Pham E.L., Bhat T., Caliari S.R. (2024). Modular Multiwell Viscoelastic Hydrogel Platform for Two- and Three-Dimensional Cell Culture Applications. ACS Biomater. Sci. Eng..

[33] Canadas R.F., Liu Z., Gasperini L., Fernandes D.C., Maia F.R., Reis R.L., Marques A.P., Liu C., Oliveira J.M. (2022). Numerical and Experimental Simulation of a Dynamic-Rotational 3D Cell Culture for Stratified Living Tissue Models. Biofabrication.

[34] Costa A.D.S.D., Vadym K., Park K. (2025). Engineered Endothelium Model Enables Recapitulation of Vascular Function and Early Atherosclerosis Development. Biomaterials.

[35] Kim K.S., Choi S.-C., Noh J.-M., Song M.-H., Jun S., Na J.E., Rhyu I.J., Lim D.-S. (2025). Validation of the Exosomal Protein SERPINA11 as a Potential Atherosclerosis Marker via Bioprinted Scaffold. Biofabrication.

[36] Echrish J., Pasca M.-I., Cabrera D., Yang Y., Harper A.G.S. (2024). Developing a Biomimetic 3D Neointimal Layer as a Prothrombotic Substrate for a Humanized In Vitro Model of Atherothrombosis. Biomimetics.

[37] Zhu J., Liu B., Wang Z., Wang D., Ni H., Zhang L., Wang Y. (2019). Exosomes from Nicotine-Stimulated Macrophages Accelerate Atherosclerosis through miR-21-3p/PTEN-Mediated VSMC Migration and Proliferation. Theranostics.

[38] Na J.-T., Xue C.D., Wang Y.-X., Li Y.-J., Wang Y., Liu B., Qin K.-R. (2023). Fabricating a Multi-Component Microfluidic System for Exercise-Induced Endothelial Cell Mechanobiology Guided by Hemodynamic Similarity. Talanta.

[39] Netala V.R., Hou T., Zhang Z. (2025). Microfluidics in Biomedicine: Heart-on-Chip Platforms for Cardiovascular Disease Modeling and Therapeutic Innovation. J. Drug Deliv. Sci. Technol..

[40] Cheng L., Chen Z., Yang F., Zheng R., He W., Shi F., Liu C., Wang F., Wang L., Xie Y. (2024). Coronary Hemodynamic Simulation Study. Proc. Inst. Mech. Eng. Part H.

[41] Chen R., Wang B., Liu Y., He J., Lin R., Li D. (2019). Gelatin-Based Perfusable, Endothelial Carotid Artery Model for the Study of Atherosclerosis. BioMed. Eng. OnLine.

[42] Shin Y., Lim S., Kim J., Jeon J.S., Yoo H., Gweon B. (2019). Emulating Endothelial Dysfunction by Implementing an Early Atherosclerotic Microenvironment within a Microfluidic Chip. Lab Chip.

[43] Jiang Y., Wang Y., Li Q., Yu C., Chu W. (2020). Natural Polymer-Based Stimuli-Responsive Hydrogels. Curr. Med. Chem..

[44] Han X., Lu B., Zou D., Luo X., Liu L., Maitz M.F., Yang P., Huang N., Zhao A., Chen J. (2023). Allicin-Loaded Intelligent Hydrogel Coating Improving Vascular Implant Performance. ACS Appl. Mater. Interfaces.

[45] Yao S.Y., Shen M.L., Li S.J., Wu X.D., Zhang M.M., Ma L.N., Li Y.P. (2020). Application of a Mechanically Responsive, Inflammatory Macrophage-Targeted Dual-Sensitive Hydrogel Drug Carrier for Atherosclerosis. Colloids Surf. B Biointerfaces.

[46] Shi P., Sun P., Lou C., Fang J., Zhang L., Xie B., Zhang C. (2025). Adventitial Injection of Hyaluronic Acid/Sodium Alginate Hydrogel Loaded with IL-33 Antibody Decreases Neointimal Hyperplasia. J. Surg. Res..

[47] Wang J., Yin G., Janghour L.M., Dai S., Akhavan B., Sun M., Zhao A. (2025). Plasma-Reinforced Dual-Crosslinked Pueraria Hydrogel Coating for Synergistic Atherosclerosis Intervention. Mater. Today Bio.

[48] Mahmoud D.M., El-Ela F.I.A., Fouad A.G., Belal A., Ali M.A.M., Ghoneim M.M., Almeheyawi R.N., Attia M.E., Mahmoud T.M. (2024). Improving the Bioavailability and Therapeutic Efficacy of Felodipine for the Control of Diabetes-Associated Atherosclerosis: In Vitro and in Vivo Characterization. Int. J. Pharm..

[49] Shang Y., Ma C., Zhang J., Wang Z., Ren C., Luo X., Peng R., Liu J., Mao J., Shi Y. (2020). Bifunctional Supramolecular Nanofiber Inhibits Atherosclerosis by Enhancing Plaque Stability and Anti-Inflammation in apoE^-/-^ Mice. Theranostics.

[50] Ma C., Feng K., Yang X., Yang Z., Wang Z., Shang Y., Fan G., Liu L., Yang S., Li X. (2021). Targeting Macrophage Liver X Receptors by Hydrogel-encapsulated T0901317 Reduces Atherosclerosis without Effect on Hepatic Lipogenesis. Br. J. Pharmacol..

[51] Chen M., Li J.-T., Gao J.-B., Zhang L., Gao Q.-H., Zeng X., Liu Q. (2025). Autologous Platelet Rich Gel in Treatment of Lower Limb Atherosclerotic Occlusive Disease in Diabetic Patients. World J. Diabetes.

[52] Mukheja Y., Sarkar A., Arora R., Pal K., Ahuja A., Vashishth A., Kuhad A., Chopra K., Jain M. (2024). Unravelling the Progress and Potential of Drug-Eluting Stents and Drug-Coated Balloons in Cardiological Insurgencies. Life Sci..

[53] Lopez-Sanchez P., Fredriksson N., Larsson A., Altskär A., Ström A. (2018). High Sugar Content Impacts Microstructure, Mechanics and Release of Calcium-Alginate Gels. Food Hydrocoll..

[54] Oaks M., Michel K., Downey F.X., Thohan V. (2018). Xenoreactive Antibodies and Latent Fibrin Formation in VAD and Cardiac Transplant Recipients Can Confound the Detection and Measurement of Anti-AT1R Antibodies. Am. J. Transplant..

[55] Zhang B., Li C., Lei D., Fan X., Wu Z., He C., Guan Q., Zhang G., Zhang P. (2025). Fast Expandable Polysaccharide-Based Cryogel Derived from Mushroom for Noncompressible Bleeding. Int. J. Biol. Macromol..

[56] Kroger S.M., Hill L., Jain E., Stock A., Bracher P.J., He F., Zustiak S.P. (2020). Design of Hydrolytically Degradable Polyethylene Glycol Crosslinkers for Facile Control of Hydrogel Degradation. Macromol. Biosci..

[57] Bramhe P., Rarokar N., Kumbhalkar R., Saoji S., Khedekar P. (2024). Natural and Synthetic Polymeric Hydrogel: A Bioink for 3D Bioprinting of Tissue Models. J. Drug Deliv. Sci. Technol..

[58] Kumar A., Sood A., Agrawal G., Thakur S., Thakur V.K., Tanaka M., Mishra Y.K., Christie G., Mostafavi E., Boukherroub R. (2023). Polysaccharides, Proteins, and Synthetic Polymers Based Multimodal Hydrogels for Various Biomedical Applications: A Review. Int. J. Biol. Macromol..

[59] Longoni A., Major G.S., Jiang S., Farrugia B.L., Kieser D.C., Woodfield T.B.F., Rnjak-Kovacina J., Lim K.S. (2024). Pristine Gelatin Incorporation as a Strategy to Enhance the Biofunctionality of Poly(Vinyl Alcohol)-Based Hydrogels for Tissue Engineering Applications. Biomater. Sci..

[60] Zhao J., Jia F., Li J., Tao Y., Hu J., Ren K., Ji J., Fu J., Fu G., Huang H. (2025). Sprayable Reactive Oxygen Species-Responsive Hydrogel Coatings Restore Endothelial Barrier Integrity for Functional Vascular Healing. ACS Nano.

[61] Rolland P.H., Mekkaoui C., Palassi M., Friggi A., Moulin G., Piquet P., Bartoli J.-M. (2003). Efficacy of Local Molsidomine Delivery from a Hydrogel-Coated Angioplasty Balloon Catheter in the Atherosclerotic Porcine Model. Cardiovasc. Interv. Radiol..

[62] Yi S., Karabin N.B., Zhu J., Bobbala S., Lyu H., Li S., Liu Y., Frey M., Vincent M., Scott E.A. (2020). An Injectable Hydrogel Platform for Sustained Delivery of Anti-Inflammatory Nanocarriers and Induction of Regulatory T Cells in Atherosclerosis. Front. Bioeng. Biotechnol..

[63] Zhu Y., Li K., Zhang Q., Nie Y., Yan T., Shi X., Han D. (2023). High-Strength Injectable Hydrogel into Perivascular Interstitial Space Enhances Arterial Adventitial Stress. Langmuir.

[64] Yang Y., Wang X., Yang F., Shen H., Wu D. (2016). A Universal Soaking Strategy to Convert Composite Hydrogels into Extremely Tough and Rapidly Recoverable Double-Network Hydrogels. Adv. Mater..

[65] Fan Z., Cheng P., Gao Y., Wang D., Jia G., Zhang P., Prakash S., Wang Z., Han J. (2022). Understanding the Rheological Properties of a Novel Composite Salecan/Gellan Hydrogels. Food Hydrocoll..

[66] Lavrador P., Esteves M.R., Gaspar V.M., Mano J.F. (2021). Stimuli-Responsive Nanocomposite Hydrogels for Biomedical Applications. Adv. Funct. Mater..

[67] Hu J., Altun I., Zhang Z., Albadawi H., Salomao M.A., Mayer J.L., Hemachandra L.P.M.P., Rehman S., Oklu R. (2020). Bioactive-Tissue-Derived Nanocomposite Hydrogel for Permanent Arterial Embolization and Enhanced Vascular Healing. Adv. Mater..

[68] Bakhrushina E.O., Novozhilova E.V., Shumkova M.M., Pyzhov V.S., Nikonenko M.S., Bardakov A.I., Demina N.B., Krasnyuk I.I., Krasnyuk I.I. (2023). New Biopharmaceutical Characteristics of In Situ Systems Based on Poloxamer 407. Gels.

[69] Milocco A., Scuor N., Lughi V., Lamberti G., Barba A.A., Divittorio R., Grassi G., Perkan A., Grassi M., Abrami M. (2020). Thermal Gelation Modeling of a Pluronic-alginate Blend Following Coronary Angioplasty. J. Appl. Polym. Sci..

[70] Wang Q., He Y., Shen M., Huang L., Ding L., Hu J., Dong Y., Fu H., Wang Q., Sun Y. (2021). Precision Embolism: Biocompatible Temperature-Sensitive Hydrogels as Novel Embolic Materials for Both Mainstream and Peripheral Vessels. Adv. Funct. Mater..

[71] Duan Y., Lin J., Yue J., Li Y., Liao J., Sun Y., Wang Q., Duan Y., Li Z. (2025). Temperature-Sensitive Hydrogel Inhibits VEGFA-Dependent Neovascularization in Atherosclerosis Progression. Sci. China Mater..

[72] Xu H., Zhao Z., She P., Ren X., Li A., Li G., Wang Y. (2024). Salvaging Myocardial Infarction with Nanoenzyme-Loaded Hydrogels: Targeted Scavenging of Mitochondrial Reactive Oxygen Species. J. Control. Release.

[73] Xing M., Bian S., Li B., Wei M., Yang Z., Li J. (2024). Functional Peptide Hydrogel for the Synergistic Treatment of Atherosclerosis Based on Macrophage Autophagy Induction and Anti-Inflammation. ACS Mater. Lett..

[74] Shao J., Liu Y., Li R., Deng Z., Liu L., Wang J., Dai S., Su Z., Cui J., Chen Y. (2025). Poly(Ethylene Glycol)-Norbornene-Heparin-Piposome Composite Hydrogels for in Situ Spraying and Ultra-Fast Adhesion: Meeting the Challenges of Endothelial Repair of Vascular Injury. Acta Biomater..

[75] Bai H., Wu H., Zhang L., Sun P., Liu Y., Xie B., Zhang C., Wei S., Wang W., Li J. (2022). Adventitial Injection of HA/SA Hydrogel Loaded with PLGA Rapamycin Nanoparticle Inhibits Neointimal Hyperplasia in a Rat Aortic Wire Injury Model. Drug Deliv. Transl. Res..

[76] Wang Y., Zhu Q., Chen Z., Wang X., Ma B., Wang Y., Xiao Y., Luo R., Zhang W., Wang Y. (2026). Self-Adaptive Covalent Coating for Vascular Stents: Coordinated Coagulation-Inflammation Regulation to Support Re-Endothelialization for Atherosclerosis Control. Biomaterials.

[77] Mann D.L., Lee R.J., Coats A.J.S., Neagoe G., Dragomir D., Pusineri E., Piredda M., Bettari L., Kirwan B., Dowling R. (2016). One-year Follow-up Results from AUGMENT-HF: A Multicentre Randomized Controlled Clinical Trial of the Efficacy of Left Ventricular Augmentation with Algisyl in the Treatment of Heart Failure. Eur. J. Heart Fail..

[78] Rao S.V., Zeymer U., Douglas P.S., Al-Khalidi H., White J.A., Liu J., Levy H., Guetta V., Gibson C.M., Tanguay J.-F. (2016). Bioabsorbable Intracoronary Matrix for Prevention of Ventricular Remodeling After Myocardial Infarction. J. Am. Coll. Cardiol..

[79] Traverse J.H., Henry T.D., Dib N., Patel A.N., Pepine C., Schaer G.L., DeQuach J.A., Kinsey A.M., Chamberlin P., Christman K.L. (2019). First-in-Man Study of a Cardiac Extracellular Matrix Hydrogel in Early and Late Myocardial Infarction Patients. JACC Basic Transl. Sci..

[80] Scotti A., Schulte M.F., Lopez C.G., Crassous J.J., Bochenek S., Richtering W. (2022). How Softness Matters in Soft Nanogels and Nanogel Assemblies. Chem. Rev..

[81] Delgado-Pujol E.J., Martínez G., Casado-Jurado D., Vázquez J., León-Barberena J., Rodríguez-Lucena D., Torres Y., Alcudia A., Begines B. (2025). Hydrogels and Nanogels: Pioneering the Future of Advanced Drug Delivery Systems. Pharmaceutics.

[82] Maddiboyina B., Desu P.K., Vasam M., Challa V.T., Surendra A.V., Rao R.S., Alagarsamy S., Jhawat V. (2022). An Insight of Nanogels as Novel Drug Delivery System with Potential Hybrid Nanogel Applications. J. Biomater. Sci. Polym. Ed..

[83] Saraogi G.K., Tholiya S., Mishra Y., Mishra V., Albutti A., Nayak P., Tambuwala M.M. (2022). Formulation Development and Evaluation of Pravastatin-Loaded Nanogel for Hyperlipidemia Management. Gels.

[84] Shoukat H., Pervaiz F., Rehman S., Akram F., Noreen S., Khan K.U., Basit A., Ashraf M.A. (2023). Development, in Vitro and in Vivo Evaluation of β-Cyclodextrin/Polyvinyl Alcohol-Co-Poly (2-Acrylamide-2-Methylpropane Sulfonic Acid) Cross-Linked Hybrid IPN-Nanogels to Improve the Dissolution and Absorption of Anti-Hyperlipidemic Drug. Polym.-Plast. Technol. Mater..

[85] Hong S., Li Y., Eom J.B., Choi Y. (2018). Responsive Alginate-Cisplatin Nanogels for Selective Imaging and Combined Chemo/Radio Therapy of Proliferating Macrophages. Quant. Imaging Med. Surg..

[86] Zhong Y., Maruf A., Qu K., Milewska M., Wandzik I., Mou N., Cao Y., Wu W. (2023). Nanogels with Covalently Bound and Releasable Trehalose for Autophagy Stimulation in Atherosclerosis. J. Nanobiotechnol..

[87] Basak S., Mukherjee I., Das T.K. (2024). Injectable Biocompatible RAFT Mediated Nitroxide Nanogels: A Robust ROS-Reduction Antioxidant Approach. Colloids Surf. B Biointerfaces.

[88] Basak S., Khare H.A., Roursgaard M., Kempen P.J., Lee J.H., Bazban-Shotorbani S., Kræmer M., Chernyy S., Andresen T.L., Almdal K. (2021). Simultaneous Cross-Linking and Cross-Polymerization of Enzyme Responsive Polyethylene Glycol Nanogels in Confined Aqueous Droplets for Reduction of Low-Density Lipoprotein Oxidation. Biomacromolecules.

[89] Zhang M., Xiao X., Li X., Shi X., Li Z. (2025). pH-Sensitive Fibronectin Nanogels Combined with UTMD for Anti-Atherosclerosis Treatment through Anti-Inflammatory and Antioxidant Effects. Mater. Today Bio.

[90] Kimna C., Miller Naranjo B., Eckert F., Fan D., Arcuti D., Mela P., Lieleg O. (2022). Tailored Mechanosensitive Nanogels Release Drugs upon Exposure to Different Levels of Stenosis. Nanoscale.

[91] Wan S., Guo Y., Liu X., Huang Y., Yao T., Wang M., Zhang Q., Wei X., Yu X., Hu J. (2025). A MicroRNA and ROS-Scavenger Co-Loaded Nanogel for in Situ Macrophage Regulation and MR-Visualized Treatment of Atherosclerosis. Adv. Healthc. Mater..

[92] Guo Y., Wang F., Wan S., Liu X., Huang Y., Xie M., Wei X., Zhu W., Yao T., Li Y. (2025). Endothelium-Targeted NF-κB siRNA Nanogel for Magnetic Resonance Imaging and Visualized-Anti-Inflammation Treatment of Atherosclerosis. Biomaterials.

[93] Huang Q., Gao H., Yang S., Ding D., Lin Z., Ling Q. (2020). Ultrastable and Colorful Afterglow from Organic Luminophores in Amorphous Nanocomposites: Advanced Anti-Counterfeiting and in Vivo Imaging Application. Nano Res..

[94] Li F., Liang Z., Ling D. (2019). Smart Organic-Inorganic Nanogels for Activatable Theranostics. Curr. Med. Chem..

[95] Wang X., Wei W., Guo Z., Liu X., Liu J., Bing T., Yu Y., Yang X., Cai Q. (2024). Organic–Inorganic Composite Hydrogels: Compositions, Properties, and Applications in Regenerative Medicine. Biomater. Sci..

[96] Zare I., Taheri-Ledari R., Esmailzadeh F., Salehi M.M., Mohammadi A., Maleki A., Mostafavi E. (2023). DNA Hydrogels and Nanogels for Diagnostics, Therapeutics, and Theragnostics of Various Cancers. Nanoscale.

[97] Cao Q.-C., Wang X., Wu D.-C. (2018). Controlled Cross-Linking Strategy for Formation of Hydrogels, Microgels and Nanogels. Chin. J. Polym. Sci..

[98] Kumar R., Parashar A. (2023). Atomistic Simulations of Pristine and Nanoparticle Reinforced Hydrogels: A Review. WIREs Comput. Mol. Sci..

[99] Wang L., Liu J., Long Y. (2018). Delayed Gelation Kinetics of Hydrogel Formation by Ionic Nano-Gel Cross-Linkers. J. Mater. Sci..

[100] Calistri S., Ciantelli C., Cataldo S., Cuzzola V., Guzzinati R., Busi S., Ubaldini A. (2025). Simple Spin-Coating Preparation of Hydrogel and Nanoparticle-Loaded Hydrogel Thin Films. Coatings.

[101] Abaee A., Mohammadian M., Jafari S.M. (2017). Whey and Soy Protein-Based Hydrogels and Nano-Hydrogels as Bioactive Delivery Systems. Trends Food Sci. Technol..

[102] Yuniarsih N., Chaerunisaa A., Elamin K., Wathoni N. (2024). Polymeric Nanohydrogel in Topical Drug Delivery System. Int. J. Nanomed..

[103] Qiu B., Cheng Q., Chen R., Liu C., Qin J., Jiang Q. (2024). Mussel-Mimetic Hydrogel Coating with Anticoagulant and Antiinflammatory Properties on a Poly(Lactic Acid) Vascular Stent. Biomacromolecules.

[104] Chen G., Chen C., Dong Z., Li X., Han C., Zhang Y., Liu B., Wen Z., Wang Y., Zhang Q. (2025). Enhancing Degradation Behaviour, Osteogenic Capacity and Angiogenic Performance of Additively Manufactured Porous Zn-Mg Scaffold via a Neobavaisoflavone-Doped GelMA Composite Hydrogel Coating. Virtual Phys. Prototyp..

[105] Chen Y., Gao P., Huang L., Tan X., Zhou N., Yang T., Qiu H., Dai X., Michael S., Tu Q. (2021). A Tough Nitric Oxide-Eluting Hydrogel Coating Suppresses Neointimal Hyperplasia on Vascular Stent. Nat. Commun..

[106] Illanes-Bordomás C., Landin M., García-González C.A. (2025). Novel Core–Shell Aerogel Formulation for Drug Delivery Based on Alginate and Konjac Glucomannan: Rational Design Using Artificial Intelligence Tools. Polymers.

[107] Carracedo-Pérez M., Ardao I., López-Iglesias C., Magariños B., García-González C.A. (2024). Direct and Green Production of Sterile Aerogels Using Supercritical Fluid Technology for Biomedical Applications. J. CO2 Util..

[108] More A.P., Chapekar S. (2024). Irradiation Assisted Synthesis of Hydrogel: A Review. Polym. Bull..

[109] Pham T.T., Tran P.L., Phung C.D., Nguyen H.T., Nguyen C.H., Yong C.S., Kim J.O., Yook S., Jeong J. (2021). Surface-Triggered In Situ Gelation for Tunable Conformal Hydrogel Coating of Therapeutic Cells and Biomedical Devices. Adv. Funct. Mater..

[110] Zhong H., Chen J., Jia S., Chen H., Xi Y., Yan Y., Lu Z., Xiao C., Xu F., Tang J. (2025). Prospective Associations of Obesity Heterogeneity, Serum Proteins, and Carotid Atherosclerosis Risk. eBioMedicine.

[111] Li W., Luo J., Peng F., Liu R., Bai X., Wang T., Zhang X., Zhu J., Li X.-Y., Wang Z. (2023). Spatial Metabolomics Identifies Lipid Profiles of Human Carotid Atherosclerosis. Atherosclerosis.

[112] Mocci G., Sukhavasi K., Örd T., Bankier S., Singha P., Arasu U.T., Agbabiaje O.O., Mäkinen P., Ma L., Hodonsky C.J. (2024). Single-Cell Gene-Regulatory Networks of Advanced Symptomatic Atherosclerosis. Circ. Res..

[113] Johnbosco C., Zschoche S., Nitschke M., Hahn D., Werner C., Maitz M.F. (2021). Bioresponsive starPEG-Heparin Hydrogel Coatings on Vascular Stents for Enhanced Hemocompatibility. Mater. Sci. Eng. C.

[114] Timoshina P.A., Surkov Y.I., Lugovtsov A.E., Priezzhev A.V., Tuchin V.V. (2025). Transmission Laser Speckle Contrast Imaging Combined With Optical Clearing Using Magnetic Resonance Contrast Agents. Lasers Surg. Med..

[115] Catoira M.C., González-Payo J., Fusaro L., Ramella M., Boccafoschi F. (2020). Natural Hydrogels R&D Process: Technical and Regulatory Aspects for Industrial Implementation. J. Mater. Sci. Mater. Med..

[116] Mohapatra S., Mirza M.A., Hilles A.R., Zakir F., Gomes A.C., Ansari M.J., Iqbal Z., Mahmood S. (2021). Biomedical Application, Patent Repository, Clinical Trial and Regulatory Updates on Hydrogel: An Extensive Review. Gels.

[117] Zhang S., Wang H., Liu F., Su Y., Han K., Liu Y., Guan F., Liu H., Ma S. (2025). Artificial Intelligence-Enabled Hydrogels: Innovations and Applications. J. Mater. Chem. B.

[118] Xu Y., Yang N., Dong S., Wang L., Qian Z., Ma R., Zhang Q., Liu Y., Chen J., Pan C. (2025). A Hydrogel Coating Mimicking the Cell Membrane for Enhancing the Corrosion Resistance and Biocompatibility of the Magnesium Alloy. Appl. Surf. Sci..

